# Isoform alterations in the ubiquitination machinery impacting gastrointestinal malignancies

**DOI:** 10.1038/s41419-024-06575-z

**Published:** 2024-03-08

**Authors:** Srimathi Kasturirangan, Derek J. Nancarrow, Ayush Shah, Kiran H. Lagisetty, Theodore S. Lawrence, David G. Beer, Dipankar Ray

**Affiliations:** 1 Departments of Radiation Oncology, University of Michigan, Ann Arbor, MI 48109 USA; 2 Surgery - Section of Thoracic Surgery, University of Michigan, Ann Arbor, MI 48109 USA; 3 Present Address: Sidney Kimmel Medical College, Thomas Jefferson University, Philadelphia, PA USA

**Keywords:** Gastrointestinal cancer, Genetics research

## Abstract

The advancement of RNAseq and isoform-specific expression platforms has led to the understanding that isoform changes can alter molecular signaling to promote tumorigenesis. An active area in cancer research is uncovering the roles of ubiquitination on spliceosome assembly contributing to transcript diversity and expression of alternative isoforms. However, the effects of isoform changes on functionality of ubiquitination machineries (E1, E2, E3, E4, and deubiquitinating (DUB) enzymes) influencing onco- and tumor suppressor protein stabilities is currently understudied. Characterizing these changes could be instrumental in improving cancer outcomes via the identification of novel biomarkers and targetable signaling pathways. In this review, we focus on highlighting reported examples of direct, protein-coded isoform variation of ubiquitination enzymes influencing cancer development and progression in gastrointestinal (GI) malignancies. We have used a semi-automated system for identifying relevant literature and applied established systems for isoform categorization and functional classification to help structure literature findings. The results are a comprehensive snapshot of known isoform changes that are significant to GI cancers, and a framework for readers to use to address isoform variation in their own research. One of the key findings is the potential influence that isoforms of the ubiquitination machinery have on oncoprotein stability.

## FACTS

- Changes in gene and protein isoform analysis are helping to better understand the development and progression of human cancers.
- Function-altering isoform variations in ubiquitination system (UBS) influence driver oncoprotein stabilities, which may be linked to cancer pathogenesis.
- While our understanding of UBS players and roles has improved substantially over the years, we don’t yet know the influences that substitutional isoforms play, particularly in the context of GI cancer.
- In the changing face of cancer therapeutics, there is a current push towards targeting protein stability as a primary or adjunct treatment option.

## OPEN QUESTIONS

- To what extent does alternative isoform availability in primary UBS genes feature in normal cellular responses to local environmental change.
- Whether protein isoform variations in the UBS play a prominent role in promoting GI malignancies.
- Whether knowledge of isoform variation can be leveraged to benefit cancer patients.

## Introduction

Cancer is a multi-step process involving cell-type specific changes in proliferation and differentiation that are highly heterogeneous. Through advances in modern genome technology, we now know that most multi-exon human genes exhibit multiple isoforms, which often (55%; [1]) result in different isoforms of the same protein. Estimating summary numbers of protein-coding isoforms per gene is dependent on factors including cell/tissue types (with many isoforms demonstrating tissue specific expression) and technical details such as read-depth, fragment technology and transcript mapping methodology [2, 3]. Consequently, estimates of the average number of isoforms per gene, or the fraction of protein coding genes with multiple isoforms vary. One estimate, based on isoforms observed in protein-coding genes within the GTEx normal tissue cohort [4] estimated an average of 7.4 transcripts per gene; however, this estimate is not exclusive to protein-coding isoforms, which we found to be 5.7 protein-coding isoforms per protein coding gene using Ensembl [5] data [105517 identified transcripts with protein status IDs/18514 genes identified as protein-coding]. In addition, proteins often exist as a family, derived from distinct genes, but with overlapping targets, cofactors, and substrates. These gene phenocopies, provide additional flexibility for biological systems to cope with environmental variation. This transcript diversity and resulting phenotypic complexity (within and between genes) is a feature of cancer causation which is intertwined with the heterogeneity of cancer biology.

In addition to protein sequence heterogeneity, most human proteins undergo post-translational modification, where a modifying group (e.g., acetyl, phosphoryl, glycosyl, and methyl) is added to specific amino acids to alter protein characteristics. The best characterized post-translational mechanism is the ubiquitin proteasome system (UPS) which regulates protein stability of a majority of intracellular proteins critical for metabolism, cell division, stress response, among other processes [6].

At the heart of the UPS is the ubiquitination machinery, consisting of five major enzyme types involved in the addition or removal of the ubiquitin moiety. ATP dependent formation of a thioester bond between an E1 ubiquitin activating enzyme and ubiquitin kickstarts the cascade [7], followed by ubiquitin transfer to a ubiquitin conjugating enzyme (E2) and finally to the target protein by means of an E3 ubiquitin ligase. The process usually works stepwise to build a ubiquitin chain that covalently attaches to the protein (most commonly through the K48 or K63 lysine residues), with K48 linkages directed for degradation via the 26S proteasome[8, 9]. There is also an enzyme category called E4 enzymes that can attach a pre-assembled polyubiquitin chain to target proteins. In addition, protein ubiquitination can be reversed via deubiquitinating (DUB) proteins. Perturbation of UPS enzymes have been implicated in various cancer types, as contributing to carcinogenic processes involving cell cycle control, cell death, migration, invasion, chemo-/radio-resistance and stimulation of key oncogenic pathways [10].

Consistent with our gastrointestinal (GI) cancer focus, this review applies a semi-automated strategy to identify within and between gene (e.g., enzyme families) isoform groups within the five ubiquitin machinery families (E1-4s, and DUBs). We summarize instances where transcriptional variation alters GI cancer biology and have drawn from TCGA cohort data using TSVdb [11] to investigate transcript diversity via minor isoform frequency assessment for each major GI cancer type. We used key word filters through the PubMed advanced interface to identify papers where E1-E4 and DUB protein-coding isoforms were associated with GI-cancer biology.

Our introduction to the influence isoform variation can have on tumor biology followed results we published while investigating how RNF128 (aka GRAIL – Gene Related to Anergy In Lymphocytes), an E3 enzyme, that regulates the protein stability of both p53 [12] and CD3 components of the T-cell Receptor Complex (TCR) [13]. Our findings on RNF128 isoforms [14] prompted us to move upstream to investigate the signaling mechanisms of the UBCH5 family of proteins that serve as E2s for UPS regulation of p53 [15] and showed that isoform specific roles within the ubiquitination machinery can be instrumental to identifying novel therapeutic interventions for GI tumorigenesis. We believe that isoform expression of other enzymes similarly involved in both ubiquitination and disease pathogenesis may also provide critical information currently lacking in the literature. Here we sequentially summarize reported isoform changes of E1-E4 and DUB enzymes, with emphasis on reported changes related to GI cancer biology.

## Methods

Details for each methods step are provided as Supplemental Methods. In summary, we focused on GI-cancer papers that discussed isoform differences for ubiquitination system (UBS) genes in association with GI cancer etiology or outcomes. We used three strategies to identify the most appropriate subset of UBS genes to discuss: gene and protein based systematic literature search for appropriate GI-cancer papers, as well as additional genes chosen based on our expertize and interests in cancer-related posttranslational modification of proteins. We justify inclusion of the latter, because systematic gene isoform investigations are relatively new in GI-cancer, whereby key isoform patterns uncovered for other cancer types may not have been studied for GI-cancers yet. From these combined approaches we compiled a list of genes, ordered by the five UPS categories. We then searched cBioportal (https://www.cbioportal.org/) GI-cancer cohort clinical data (sub-categorizing samples base on clinical histology types) and TSVdb (http://www.tsvdb.com/instruction.html) isoform data. From the resulting compilation we determined isoform frequencies for all cancer pathology groups with 10 or more samples, as well as for the 160 non-cancer tissues from cancer patients present across GI-cancer cohorts. We used these data to estimate diversity for each gene, in each cohort, via three methods: Shannon Diversity Index, Shannon Equitability Index and minor isoform frequency. We found that for all but a few genes (discussed below) minor isoform frequency was a good approximation for the other diversity measures, using Pearson correlation. As the simplest and most direct measure, we chose to present minor isoform frequencies in the manuscript. Lastly, we contrasted minor isoform frequency for each gene, in each cancer pathology group, against those found in the compilation of non-cancer tissues from GI-cancer patients. Identified disparities represent genes where cancer-specific changes in protein-based isoforms should be investigated for relevance to cancer etiology/outcomes.

## Isoform variation in Ubiquitination System (UBS) in esophageal cancer

### Ubiquitin Activating (E1) Enzymes

Human cells express only two ubiquitin activating enzymes (E1): *UBA1* and *UBA6*, which pair with over 40 different ubiquitin conjugating enzyme (E2) family members to initiate the sequential process of ubiquitination. Due to its key involvement in energy dependent activation of the ubiquitin moiety to initiate the process of protein modifications of all target proteins irrespective of type (mono- versus poly-ubiquitination) and linkages (K48, K11, K63 etc.), E1 targeting was initially feared to bring non-specific toxicity [16]. However, the clinical success of bortezomib (proteasome inhibitor), and preclinical testing of TAK-243 (E1 inhibitor) provided evidence for limited toxicity yet therapeutic advantage in controlling leukemia, suggesting that an improved understanding of ubiquitination machinery activation could have significant translational potential.

#### UBA1

Since its discovery in the early 1980s, the broad cellular distribution and functionality of X-chromosome linked UBA1 has been well documented [17, 18]. While an essential mammalian protein, dysregulation of UBA1 via mutationally impaired functionality has been linked to serious, syndrome-like ailments, which symptomatically overlap with Spinal Muscular Atrophy [19]. Part of the complexity arises because UBA1 has two normally expressed protein isoforms resulting from alternate methionine initiation sites (position 1; UBA1a which typically has a nuclear localization and position 41; UBA1b, typically a cytoplasmic localization) [20]. In a recent study, Beck et al. identified a rare somatic, but postzygotic, mutation of methionine 41 in UBA1 that was concentrated in myeloid cell-types and resulted in a mutant-specific version of isoform UBA1b initiated from an alternate methionine (position 67). This N-terminal truncated short form was found to be enzymatically inactive, while the M41* carrying, full-length UBA1a form was still active. Expression of the truncated UBA1b isoform resulted in cellular stress due to unfolded protein response (UPR) activating the innate immune system and autoimmunity [21]. Despite its pathological relevance, here we have avoided inclusion of isoforms for genes generated following accumulation of mutations.

**Fig. 1:**
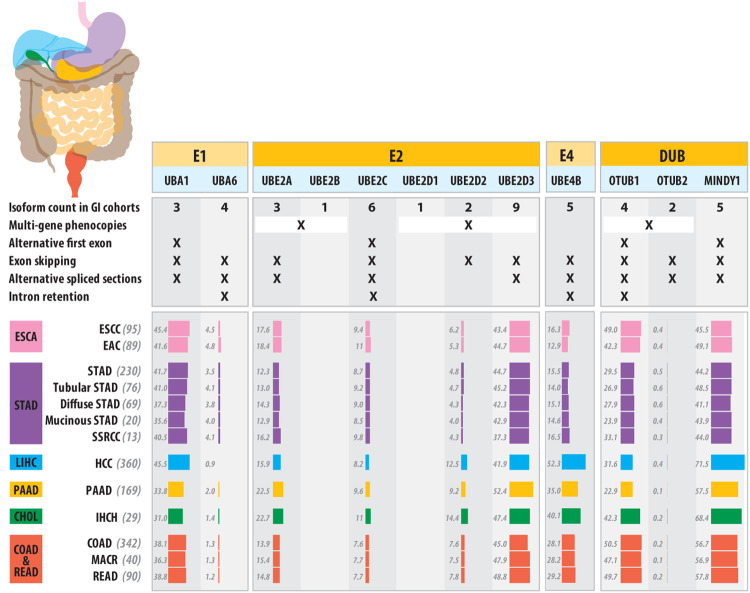
Showing E1, E2, E4, and DUBs gene symbols [protein or previous common symbol] for loci groups we found to have within or between-gene isoform pattern differences in TCGA GI-cancer cohorts or literature. The figure includes vertical sections which indicate isoform structural differences seen for each gene, followed by minor isoform frequencies (collated via the TSVdb webtool) for GI cancer cohorts, where key (those with >10 samples) tumor types are presented, ordered and color-coded based on the organ in which they arise (ESCA - esophagus [pink]; STAD - gastric/stomach [purple]; LIHC - liver [blue]; PAAD - pancreas [yellow]; CHOL - bile duct [green]; COADREAD - colon and rectum [red]), with cancer cell-type abbreviations as used by TCGA and ranked by tumor sample numbers within each cohort. Values represent minor isoform frequency, as a simple measure of transcript diversity. For all genes in Fig. 1 minor isoform frequencies across tumor types showed greater than 0.75 Pearson correlation to both the Shannon Diversity Index and the Shannon Equitability Index (the latter being less sensitive to sample size).

Limited information is available regarding isoform specific UBA1 expression or functionality in GI cancer types, probably due to its near universal expression, its non-specific role in UPS and X-linkage. Zhang and coworkers did identify significant alternative *UBA1* splicing in colorectal cancers (CRC), as compared to healthy colon tissue, although no clinical variations were attributed to the different spliced forms [22]. In hepatocellular carcinoma (HCC), upregulation of UBA1 was found to be oncogenic via initiation of *NRF2* signaling, which led to reduced ferroptosis enabling cells to survive and promote HCC [23]. These researchers suggested *UBA1* expression may serve as a prognostic marker in HCC, though isoform variation was not investigated [23]. In Fig. 1, we show that the two minor *UBA1* isoforms seen in TCGA GI-cancer cohorts including HCC, are both present in relatively high frequency, with minimal variation between cancer types. We saw no significant differences in isoform frequencies in either HCC or CRC when compared to organ specific control tissues (analyzed published data, not shown). The most common of the two minor isoforms (*UBA1b*) utilizes an alternative first exon resulting in a shorter protein without a nuclear localization signal [24], compared to the common isoform. The remaining and rarest *UBA1* isoform is severely truncated missing the initial 17 exons and beginning with an alternately spliced 18^th^ exon. Pathological significance of such isoform variations remains unknown.

#### UBA6

Only confirmed as an alternative E1 for UPS in 2007 [25], UBA6 was found to be as critical to UBA1 for human cell proliferation, with an overlapping, but distinct E2 partner and substrate profile. Like UBA1, UBA6 is involved in initiating the ubiquitination process, although it diverges from UBA1 via a role in protein degradation through the ubiquitin-like modifier FAT10 [26, 27]. In breast cancer, 38% of invasive carcinomas have downregulated UBA6 perhaps because activation of this ubiquitin pathway created a suppressive barrier against vital mammary carcinogenesis processes involving the loss of polarity, anoikis resistance, and epithelial-mesenchymal transition [28]. Similarly, UBA6 expression is downregulated in bladder cancer [29]. In GI cancers very little information is available on UBA6 isoforms. In TCGA GI-cancer cohorts, we found evidence of five *UBA6* isoforms, with the primary, full-length version accounting for more than 95% of cohort-averaged expression in each major cancer type, while each of the other four isoforms present severely shortened protein forms. There is little opportunity for these rare variant isoforms to contribute to disease burden (Fig. 1).

### Ubiquitin Conjugating (E2) enzymes

The E2 enzymes are more substrate specific as compared to E1s, with more than 40 E2s expressed in humans. Some E2s have the capability to interact with both UBA1 and UBA6 while others are restricted to one or the other. Clusters of E2s each fulfill a subset of functions (via substrate specificity) ascribed to E1s, but with overlapping functionality within and particularly between E2 families. Certain E2s function in pairs (as heterodimers) to complete their role in the ubiquitination process (e.g., UBE2V-UBE2N), while a few others (e.g., CDC34) function as homodimers. Here, we have summarized selected E2 families that undergo isoform alteration, potentially contributing to GI oncogenesis.

#### RAD6 (UBE2A/UBE2B)

The RAD6 protein has two isoforms (RAD6A and B) with 95% protein sequence identity that are encoded by the *UBE2A* (X chr.) and *UBE2B* (5q31) genes respectively. In TCGA GI cancer cohort data, we found three *UBE2A* isoforms and a single *UBE2B* mRNA form (Fig. 1). This contrasts with melanoma, where a greater diversity and upregulation of RAD6B transcripts were detected as compared to normal melanocytes [30]. RAD6 is overexpressed in other cancers including ovarian [31], breast [32, 33] and colon [34] with links to chemoresistance [33] and patient survival [34], however the potential role of isoform variation remains unknown.

#### UBCH10 (UBE2C)

UBCH10, encoded by the *UBE2C* gene, regulates cell cycle via controlling cyclin expression to influence cyclin dependent kinase (CDK) activity. UBCH10 partners with the anaphase promoting complex/cyclosome (APC/C) to degrade mitotic cyclin (Cyclin B) to influence mitotic exit/progression. In high-grade astrocytoma, overexpression of UBCH10 is linked to aneuploidy and tumor formation [35]. A meta-analysis of pan-cancer TCGA data found *UBE2C* up-regulation in a majority of tumor types, relative to normal tissue, and higher expression was associated with advanced stage and poor survival [36]. These data support previous cohort-specific studies including esophageal and hepatic cancer cases [37–40] and may be linked to higher proliferation rates [41]. *UBE2C* has six isoforms (v1-6) found in gastrointestinal cancers (Fig. 1). Among them, isoform v1 (the most commonly expressed form) is upregulated in malignant compared to normal tissues [36] leading to premature override of the spindle assembly checkpoint and aberrant mitosis causing aneuploidy and uncontrolled cell growth. Our isoform diversity analysis confirms isoform v1 as the most prominent across GI cancer types, with the other 5 isoforms collectively representing around 10% of cohort expressions (Fig. 1).

#### UBCH5

The *UBE2D* gene family consists of four members of UBE2D1-D4 corresponding to UBCH5A, B, C, and D protein isoforms. Among vertebrates UBCH5s are universally expressed with high inter-family homology (~90%) but different chromosomal locations. UBCH5 proteins are involved in multiple cellular signaling events, including those for receptor tyrosine kinases, Hedgehog, TGF-β, and NF-κB, leading to modulations in chromatin-related processes including DNA methylation, DNA repair and histone modifications [42]. Members of UBCH5s differentially influence the stability of p53 in unstressed cells; while UBCH5A knockdown had minimal impact on p53 levels, loss of UBCH5B and UBCH5C resulted in increased p53 stability [43]. The gene for UBCH5A (*UBE2D1*) and that of UBCH5C (*UBE2D3*) exhibited inverse expression patterns in esophageal tissue samples, such that UBCH5A had higher expression in esophageal adenocarcinomas (EACs) compared to non-dysplastic Barrett’s esophageal (NDBE) samples, with the opposite seen for UBCH5C levels, potentially via copy number loss [15]. Given that mutant p53 is often stabilized and contributes to tumorigenesis for most GI cancers including >70% of EACs, these findings indicate how understanding the isoform specific roles of ubiquitination machinery could be instrumental in identifying novel therapeutic targets to prevent GI tumorigenesis. Importantly, while high UBCH5C expression was associated with better prognosis in esophageal cancer, increased UBCH5A was shown to be an unfavorable prognostic implication in lung cancer [44, 45]. Cadmium poisoning causes nuclear p53 stabilization and nuclear accumulation in a dose dependent manner was linked to the changes in the UBCH5 family members [46]. These studies are suggestive of *UBE2D* isoform changes as a common underlying mechanism for various pathological states.

### Ubiquitin Ligase (E3) enzymes

By far the largest class (>600 members) of UPS enzymes are involved in the process of tagging substrates with the ubiquitin moiety. These E3 ubiquitin ligases tend to be highly specialized towards dedicated substrate subsets, distinguishing them from E1, E2 and DUB classes. This mega-family of enzymes is subdivided based on structural domain types into (i) RING (Really Interesting New Gene), (ii) HECT (Homology to E6AP C-terminus) and (iii) U-box containing ligases [47, 48] then further divided based on substrate recognition and sequence homology into particular families.

#### RING family members

##### RNF128/GRAIL

Our introduction to the influence that isoform variation can have on tumor biology followed results we collected with RNF128 (aka GRAIL – Gene Related to Anergy In Lymphocytes), an E3 enzyme involved with the stability of p53, as well as CD3 components of the T-cell Receptor Complex (TCR) [13]. In a cohort of columnar-enriched tissue samples that spanned the progression of non-dysplastic Barrett’s esophagus (NDBE) through histological intermediates of low-grade (LGD) to high-grade dysplasia (HGD), and finally to EAC, we noticed increased transcriptional activity within spliceosome machinery genes. We correlated this spliceosome activity change to an increase in overall transcript diversity. We speculated this to be an ATM signaling-driven stress-response whereby pressure from acid/bile reflux and chronic inflammation, as well as goblet cell and mucin loss (hallmarks of dysplastic progression), lead to higher levels of DNA damage in the remaining (dysplastic) columnar cells. Examining which genes increased spliceosome activity perturbed the most, via isoform imbalance, we identified *RNF128* which exhibited a pronounced isoform ‘switch’ during progression. An almost universal reduction in RNF128-Iso2 was present in our cohort of HGD and EAC samples, relative to NDBE and LGD, while the mRNA level of Iso1 remained relatively constant, leading to a switch in most predominant isoform. Increased protein levels of Iso1 were found in HGD/EAC cell lines and patient tissues which correlated with nuclear p53 accumulation, and with the presence of *TP53* mutation. Additional experimentation confirmed [14] that, in esophageal columnar cells, Iso1 fulfills a protective role where it stabilizes p53 and mutant p53 while Iso2 promotes the poly-ubiquitination and degradation of these proteins. It is worth noting that this function of Iso1 is crucial to EAC development since knockdown of mutant p53 results in cell death. This offers tremendous potential for tissue-specific therapeutics as new molecular, isoform-based targets that can be explored to treat patients. The isoform expression of *RNF128* can serve a dual purpose in diagnosis as well through the analysis of the ratio of Iso1 to Iso2 to determine the severity of the progression [14].

##### MDM2

**Fig. 2:**
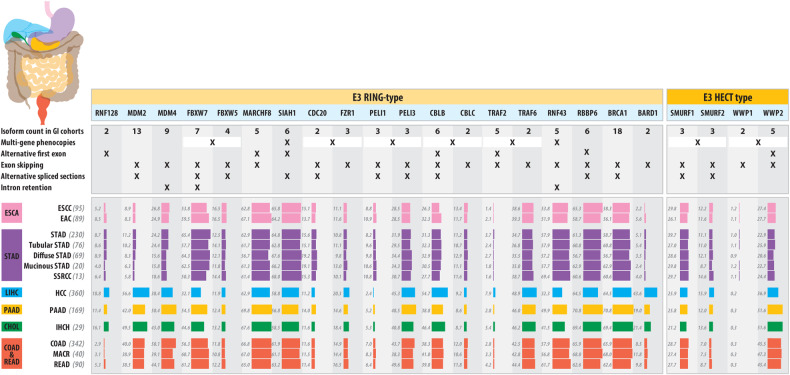
Showing E3 gene symbols clustered by RING and HECT subtypes, with loci sub-clustered based on within or between-gene isoform pattern differences in TCGA GI-cancer cohorts or literature. The figure includes vertical sections with indicate isoform structural differences seen for each gene, followed by minor isoform frequencies (collated via the TSVdb webtool) for GI cancer cohorts, where key (those with >10 samples) tumor types are presented, ordered and color-coded based on the organ in which they arise (ESCA - esophagus [pink]; STAD - gastric/stomach [purple]; LIHC - liver [blue]; PAAD - pancreas [yellow]; CHOL - bile duct [green]; COADREAD - colon and rectum [red]), with cancer cell-type abbreviations as used by TCGA and ranked by tumor sample numbers within each organ. Values represent minor isoform frequency, as a simple measure of transcript diversity. All genes, other than those indicated in the footnote, showed greater than 0.75 Pearson correlation between minor isoform frequencies across tumor types, versus either the Shannon Diversity Index or the Shannon Equitability Index (the latter being less sensitive to sample size). *Indicates markers where the minor isoform frequency correlation (Pearson) to either Shannon Diversity index was less than 0.75. The worst of these was BRCA1 and MDM4, where minor isoform frequency to diversity index Pearson correlation co-efficient was below 0.4 for each, as well as PELI3 and RBBP6 (correlations below 0.7) and SIAH1 (correlations between 0.7 and 0.75 for both Shannon diversity metrics).

Aberrant MDM2 expression induces tumorigenesis [49]. Relationships between MDM2 isoforms and p53 have proven to be complex and have been reviewed extensively [50]. Given that there are many MDM2 isoforms (we noted 13 in GI cancer cohorts, but not all seen in each tissue type) that differ in subcellular localization [51], normal verse tumor tissue-type profiles [52], response to external stimuli like tobacco usage [51], substrate interactions [53, 54], ubiquitination potential [55, 56] and cancer mutation profile patterns. A further complication is the multimodal relationship between MDM2 and MDM4 (each with many isoforms) relative to p53 stabilization and the role isoforms may play in that [56]. The *MDM2* is reported to be amplified in gastric cancer without information on potential isoform changes [57]. While analyzing our data pertaining to BE to EAC progression, levels of *MDM2* were found to be low with no significant shifts in *MDM2* isoform profiles [14]. When we consider pan-GI cancer cohorts, however, a striking shift in the relative isoform diversity is apparent for *MDM2* and, to a lesser extent, *MDM4* (Fig. 2). These data indicate that the GI cancer initiating north of the colon (ESCC, EAC and STAD as the major forms) show low levels of alternate isoforms (less than 15%; Fig. 2) while cancer types below this point show a distinctly higher level of isoform variation (39% or higher). This suggests that alternative *MDM2* isoforms are more likely to be disease-relevant in lower GI cancers. The reason for this dichotomous shift in *MDM2* isoform diversity is unknown, but *MDM4* shows a similar, though less pronounced, shift (Fig. 2).

##### F-box and WD containing E3s

FBXW7 is a F-box family member and part of the Skp1/Cullin/F-box protein (SCF) complex. FBWX7 and other F-Box proteins recognize substrates via the CDC4 phosphodegron motif which requires target proteins to be phosphorylated to promote ubiquitination dependent degradation [58]. While FBXW7 and other FBXW family members are either mutated or undergo copy number change in different cancers, interest arises when both an FBXW member and an oncogenic target protein are alternately perturbed, resulting in accumulation of the activated (phosphorylated) oncoprotein [58–60]. The best-studied example is the mutational partnering between FBXW7 and NOTCH1 mutations that collectively account for more two thirds of adult onset of T-cell acute lymphoblastic leukemia (T-ALL) cases ([61]; reviewed by [62]) resulting in NOTCH1 signal activation [63]. While both copy number loss (4q31) and mutation in FBXW7 are present in 20 to 50% of GI cancer types (ESCC [64], EAC [65, 66], STAD [67, 68], CRC [59, 69]) marking it as a key tumor suppressor, the specific targets (which include c-MYC, Cyclin E, AURKA, mTOR, JUN and NOTCH1 [70]) are less distinct, and possibly multifactorial. A similar association between FBXW4 and cyclin D1 was noted in ESCC [71]. Another FBXW7 cancer association [70] is the reporting of an inverse correlation between reduced FBXW7 level and p53 mutation status. p53 is a known target of FBXW7 mediated degradation [72], and, in contrast, *FBXW7* expression is regulated by p53 [73], which is compromised following mutations in several GI cancers [74, 75].

*FBXW7* generally expresses three main protein-coding isoforms (α, β, and γ) that differ in subcellular localization [76]. In GI cancers, we noted a total of seven *FBXW7* isoforms, with consistently high minor isoform diversity (32–65%) (Fig. 2), suggesting that isoform variation should be taken into consideration when assessing *FBXW7* expression. This contrasts with co-F-box family members *FBXW5* and *FBXW4* where we noted five and a single isoform, respectively. Furthermore, the consistently lower diversity of *FBXW5* isoforms (10 to 20%) suggests they represent relatively minor sources of expression variance (Fig. 2).

The overexpression of SCF complex scaffold-forming members of the Cullin family (particularly Cullin 1 and 2) have also been noted in GI cancer types [77–79], and this forms a further source for heterogeneity in cancer-related FBXW activity. Further understanding of the role of such isoform variation may improve knowledge in the development and progression of GI cancer cases.

##### APC/C

Anaphase promoting complex/cyclosome (APC/C) is also a multi-subunit E3 ligase that is conserved from yeast to humans, and primarily functions to facilitate metaphase to anaphase transition during mitosis. It contains ~13 different subunits that form a tetratricopeptide repeat lobe and a catalytic core. The catalytic core triggers polyubiquitination and 26S proteasomal degradation of substrates involved in cell cycle regulation [80]. APC/C regulates G1 and M phases in the cell cycle by binding to either one of its coactivators CDC20 or its homolog, FZR1 (formerly CDH1), with each contributing to APC/C ligase substrate specificity via WD40 repeat domains. CDH1 is reported to express splice variants alpha (α) and beta (β). Both CDH1α and β can associate with the APC subunits to regulate Cyclin B1 stability. CDH1α encodes a nuclear localization signal (NLS) which is absent in CDH1β, hence the β isoform is predominantly cytosolic. In *Xenopus* embryos, overexpression of CDH1α can down-regulate Cyclin A in the nucleus to induce G1 arrest, but CDH1β does not. This suggests functional variation between the two isoforms, and similar isoform changes may happen in cancers, though this is currently unknown [81]. In TCGA GI cancer cohorts, we noted two and three isoforms for CDC20 and FZR1, respectively. While minor isoforms for each of these genes represent less than 20% cohort expression (Fig. 2), the very small number of minor isoforms suggest they should be considered as part of an association investigation to either of these genes.

##### Pellino Family (RING like)

PELI E3 ligases were initially identified as Interleukin-1 receptor associated kinase (IRAK) interacting proteins and are activated by IRAKs or TBK1, to facilitate K63-linked ubiquitination of kinases. As scaffolding proteins, Pellino family members interact with wide range of pattern recognition receptor (PRR) signaling molecules and function as E3 ligases [82]. There are three family members, each having distinct functions. PELI1 ubiquitinates RIP1 and activates NF-κB signaling. Phosphorylation of PELI1 by IRAK1 and IRAK4 greatly enhances its ligase activity to promote K63 linked polyubiquitination of IRAK1 to aid NF-κB activation.

PELI1 is also important in MYD88 mediated MAPK signaling in neuro-inflammation and is a negative regulator of T lymphocytes. PELI2 is also phosphorylated by IRAK1, promoting K63 polyubiquitination of IRAK1, which contributes to the degradation of IKK to activate the NF-κB pathway [83]. PELI2 is also important in NLRP3 inflammasome signaling required for the processing of pro-IL1β and IL18. In contrast, PELI3 ubiquitinates TRAF6 and targets RIP1 and RIP2 kinases [84, 85].

In GI cancer, we noted multi-isoform expression for PELI1 and PELI3, each with three isoforms (Fig. 2) PELI1 had low minor isoform frequencies, while the minor isoforms to PELI3 were seen at higher frequency, suggesting potential for a role in functional perturbation.

##### CBL

The Casitas B lineage lymphoma (CBL) subfamily consists of three proteins CBL, c-CBL and b-CBL. These proteins are key regulators of cell signaling; c-CBL can mediate adapter like functions and interacts with several cytoskeletal proteins that influence cell invasion, motility and adhesion, while b-CBL negatively regulates natural killer cell function and CD8 T-cell stimulation [86, 87]. Human CBL negatively regulates EGFR signaling and contains both a RING finger domain and a proline rich region. In Drosophila, Cbl exists as two distinct isoforms, D-CblS (lacks proline rich region) and D-CblL (contains both RING finger and proline rich regions). These isoforms serve to internalize and degrade EGFR, yet D-CblL, but not D-CblS, and can effectively rescue constitutive EGFR signaling when overexpressed. D-CblL localizes to the cytoplasm while D-CblS is found at the follicle cell cortex suggesting distinct mechanisms of action [88]. c-CBL is mutated in several human solid tumors, and mutant c-CBL protein behaves as a dominant negative, binding to EGFR preventing its ubiquitination by wildtype c-CBL. Loss of c-CBL is associated with myeloid neoplasms [89]. c-CBL undergoes exon skipping in glioma cells resulting in the formation of two isoforms: type I (lacking exon 9) and type II (lacking exon 9 and 10). These isoforms undergo rapid proteasomal degradation leading to prolonged EGFR activation in rat C6 and human A172 cells, consequently resulting in malignancy. c-CBL exon skipping occurs in brain cells grown under hypoxic or high-density conditions and is linked to glioma development suggesting further investigation is required to understand the complex signaling pathways that determine the role of CBL family of proteins in cancer progression [90].

Figure 2 shows that CBL had six different isoforms expressed in GI cancer, with minor isoforms having high, and quite variable expression across GI cancers: highest in liver and lowest in esophageal cancers. We noted changes in the most frequent isoform between cancer types (data not shown), a further suggestive indication of interest.

##### RBBP6

RBBP6 (Retinoblastoma binding protein 6) contains a Ring finger, a zinc knuckle, and a ubiquitin like DWNN domain. RBBP6 has four known isoforms with distinct functions and that are associated with spliceosome regulation via distinct target proteins [91]. Isoform 1 interacts with tumor suppressors p53 and RB and can ubiquitinate p53, suggesting a role in regulating the cell cycle and apoptosis. Isoforms 1 and 2 are structurally similar with each containing all three key domains, while isoform 3 contains only the DWNN domain. Isoform 3 expression regulates apoptosis and functions as a tumor suppressor. Isoform 4 does not have the Rb binding domain and is poorly studied [92]. *RBBP6* is known to be upregulated in esophageal cancer [91]. Immunohistochemistry data in normal and cancerous colon tissues revealed that RBBP6 isoform 3 was linked to induction of apoptosis and decreased BCL2 expression. Isoform 1 was also found to be upregulated in colon cancer tissue [93]. RBBP6’s interactions with RB and p53 mark it an important genetic target and potential marker for cancer diagnosis. RBBP6 had six isoforms in GI cancers, with consistently high minor isoform frequencies across all cancer types, indicating potential for a pan-cancer isoform influence on RBBP6 mRNA or protein.

##### BCRA1 and BARD1

Breast Cancer 1 (BRCA1) and BRCA 1 associated Ring domain 1 (BARD1) are E3 ligases that form heterodimers that function as tumor suppressors and are predominantly mutated in breast cancers [94]. A study using nanopore sequencing of full length BCRA1 mRNA transcripts revealed 32 complete isoforms, including 18 novel forms resulting from skipping of multiple contiguous and noncontiguous exons. Most isoforms of BRCA1 result from combining alternative splice junctions, and very little is known about each isoform and its function [95]. The *BRCA1* gene variants can have similar function to BRCA1 full length protein or oppose its function and must be studied in a context specific manner to provide more insights [96]. Recently, differential expression of multiple BARD1 isoforms, that do not contain the BRCA1 binding domain, have been detected in colon and non-small cell lung (NSCLC) cancers [97, 98]. When normal tissues and NSCLC samples were analyzed to identify isoforms of BARD1, a novel isoform π was found to be upregulated in tumors [97]. Cancer specific regulation of splice variants may be essential to understand how each variants impact progression, however the ratio of full-length BARD1 to splice variants could be a prognostic marker in colon cancer [99]. Using TCGA GI cancer cohorts, we saw two protein-forming isoform mRNA species for *BARD1*, but a total of 19 for *BRCA1* (Fig. 2). The minor isoforms for *BRCA1* represent a good proportion of average sample expression in all 13 major GI cancer types present in TCGA data, in fact collectively more than the main isoform in each case (Fig. 2). For BARD1, this was only true for HCC, and to a lesser extent IHCH and PAAD, suggesting that minor allelic forms are of potential interest in liver, bile duct and pancreatic cancers, but not the other GI-cancer forms. Using a supplemental analysis which tallies the most expressed isoform for each sample, we find that, among normal samples (*n* = 160), the HCC (*n* = 50) subset was evenly split with regards to the most common isoform, while other main control groups (ESCA *n* = 11, STAD *n* = 35 and COADREAD *n* = 51) all showed strong bias (>90%) towards isoform uc002ozs being the most prominent (data not shown). These data suggest that the strong BARD1 isoform variability in HCC, originates from, founding cells with a high expression of both isoforms (unlike in esophageal, gastric and colon cancers), which transfers to tumors. Thus, an understanding of this difference should begin with why normal liver (versus other GI tissue) and would feature both protein isoforms. It is of interest to note that the two BARD1 isoforms observed in TCGA GI cancer data represent full length (uc002veu via NCBI and BARD1-201 via ENSEMBL; a 777 aa protein predicted) and lacking exon 2 (uc010zjm via NCBI and BARD1-212 via ENSEMBL; 758 aa protein predicted) isoforms, the later representing a truncated RING-domain. Other isoform versions, including those lacking more substantial portion central to the protein [99, 100], did not gain any reads.

#### HECT Family Members

##### SMURF1 and SMURF2

These are homologous to the E6AP C-terminus (HECT) family E3 ligases initially identified as critical players in the transforming growth factor beta (TGF-β) and bone morphogenetic pathway (BMP) regulators. SMad Ubiquitination Regulatory Factor 1 and 2 (SMURF1 and SMURF2) are involved in cell proliferation and differentiation, chromatin organization and dynamics, DNA damage response and genomic integrity maintenance, gene expression, cell stemness, migration, and invasion and are reviewed elsewhere [101, 102]. SMURF1 aids in the ubiquitination and degradation of SMAD proteins (SMAD1, 5 and 8) as a part of the bone morphogenic pathway (BMP), while activating SMAD2 and SMAD3 contributing in TGF-β signaling [103]. SMURF1 is widely considered an oncoprotein whose overexpression was detected in 68 gastric cancer samples when compared to adjacent normal tissue samples. For this cancer, SMURF1 targets DAB2IP and its restoration could inhibit gastric cancer cell proliferation and invasion [102, 104]. In contrast, oncogenic potential of SMURF2 is tissue type specific; SMURF2 regulates MAD2 protein stability, a key player in spindle assembly checkpoint and mitosis progression [105], and is involved in stabilizing EGFR and mutant KRAS protein stability in lung cancer. Hence, SMURF2 is considered as an oncoprotein [106, 107]. In contrast, in fibroblasts, SMURF2 is considered to be a tumor suppressor involved in maintaining genomic integrity by regulating RNF20 levels [108]. SMURF1 has 2 isoforms, produced by alternative splicing; namely SMURF1-L (long), SMURF1-S (short) [101]. SMURF1’s isoforms have high sequence homology, bind to the same substrates and have similar structures, but could have distinct biological activity [109]. A splice variant of SMURF2 (ΔE2) is overexpressed in CD4^+^ T cells that can degrade wildtype SMURF2 to reduce proliferation, instead it promotes production of proinflammatory cytokines (IL6). Consequently, this isoform variant of SMURF2 protects mice against colitis associated colon cancer [110]. Across GI cancer types *SMURF1*, but not *SMURF2*, showed moderate minor isoform expression (Fig. 2).

##### WWP1 and WWP2

WWP1 gene copy number gain is found in 30–50% of cancers and it is overexpressed in about 60% of prostate and breast cancer samples, where it promotes ubiquitination and degradation of the p63 tumor suppressor protein [111]. WWP1 can also complex with wild type p53 to enhance stability, but that facilitates nuclear to cytosolic translocation reducing p53 transcriptional activity [112]. The WWP1 gene is localized on 8q21 and alternatively spliced to generate at least six different isoforms that diverge structurally in the N-terminal C2 domain. These isoforms are expressed in a tissue specific manner and can regulate ligase activity [113]. WWP1 can also heterodimerize with WWP2 to selectively degrade the truncated p73 isoform essential for cell survival. WWP2 selectively ubiquitinates and degrades p73, and WWP2 is inactivated in conditions of cellular stress, upregulating p73. WWP1/2 dimers degrade the dominant negative truncated p73 protein, supporting proliferation and cell survival [114]. WWP2 has 3 splice variants, WWP2-FL, C and N (full length, C and N terminal). These isoforms are differentially expressed in different cancer types and can regulate SMAD2/3 turnover to either promote EMT or act as tumor suppressors [115]. Studying isoforms of WWP2 and WWP1/2 heterodimerization in gastrointestinal cancer could prove instrumental in developing novel means of targeting these cancers. WWP2 showed a similar minor isoform profile to MDM2 and MDM4, and upper GI-cancer types (those of esophagus and stomach) showed weak minor isoform expression, as compared to those below. This trend was very similar in strength to MDM4, and, interestingly, MDM4 is known to bind p73 and p63 with a higher affinity than that of MDM2 [116]. So, potentially this isoform pattern which we do see reflected in non-cancer GI tissue (data not shown) is somehow related to tissue-based differences in p73/p63 utilization [117].

#### Other E3 ligases with limited isoform information

Analysis of available datasets revealed the existence of isoforms for several other E3 ligases. There is little to no information on isoform specific studies for these E3s, and further research could provide valuable insight into their respective functions in tumorigenesis. For example, MARCH 8 (Membrane associated Ring-CH; now using gene symbol *MARCHF8*) is a part of the MARCH subfamily of E3 ligases that contains 11 members. Increased *MARCH 8* mRNA and protein levels were found in 86% of esophageal squamous cell carcinoma (ESCC) samples when compared to distant non-malignant tissues [118] and also in gastric cancer cells [119]. MARCH 8 plays an important role in cancer progression and could potentially have five isoforms (Fig. 2).

SIAH family with three family members (SIAH1-3) has an N-terminal RING finger, two zinc finger, and a C-terminal substrate binding domain (SBD) [120]. The family members have overlapping substrate specificity but are expressed under divergent conditions. SIAH1 is induced upon DNA damage in a p53-dependent manner, whereas SIAH2 is ubiquitously expressed in hypoxic cells. SIAH1 is considered as a tumor suppressor, while SIAH2 is possibly a proto-oncogene [121]. From the TCGA dataset we have identified six isoforms of SIAH1 (Fig. 2) with limited information on functional differences [122]. Both *MARCHF8* and *SIAH1* showed the presence of multiple isoforms (5 and 6 respectively), with very strong minor isoform frequency representation across GI-cancer types (Fig. 2). For both genes, more detailed investigation of isoform expression patterns demonstrated several prominent isoforms that shift their patterns of transcript representation in a roughly tissue-specific manner, including within available non-cancer samples.

The TRIM (Tripartite motif) family is the largest family of RING finger containing E3 ligases and consist of 11 members. TRIMs contain an N terminal RING finger domain, two zinc finger domains and a coiled coil region along with a diverse C-terminal domain. This group of proteins regulates a variety of cellular functions including metabolism, cell transformation, stem cell renewal, gene transcription, proliferation and can have both oncogenic and tumor suppressive roles [123]. For the *TRIM* gene family, we did not identify multiple isoforms (Fig. 2).

The TRAF (TNF receptor associated factors) subfamily consists of six classical proteins [1–6] that contain the TRAF amino acid homology domain, which is absent in TRAF7. TRAFs are essential for immune receptor signaling, cell proliferation, differentiation, survival, and apoptosis via TLR and MAPK signaling pathways. TRAF1, 2, 4, 5 and 6 are potential oncogenes while TRAF3 could act as a tumor suppressor [124, 125]. The functions of the specific isoforms and their possible role in lymphoma, other cancers, and inflammation are poorly understood. For TRAF6, we noted two isoforms, and both are well-used (Fig. 2).

### E4 ligases (Ubiquitin chain elongators)

E4 consists of a family of proteins that work in conjugation with E1, E2 and E3 enzymes and aid in elongating the ubiquitin moieties added to target proteins. E4s thereby enhance polyubiquitination efficiency and effective proteolysis [126, 127]. E4s can add a polyubiquitin chain directly onto proteins targeted for degradation (K48 linked) as opposed to the repeated addition of monoubiquitin molecule by E3 ligases, fast tracking proteasomal degradation often essential to regulate stress and oncogenesis [126, 128–130]. Several E3s can act as E4 ligases or cofactors to the multiubiquitination. However it is unclear if E4s are specific to distinct E3s [130, 131]. There are several enzymes including UBE4B/UFD2a, p300/CBP. BUL1/2, CHIP/STUB1, RNF11, Gp78 etc., which have been reported to have E4 activity with known targets for some [132].

#### UBE4B/UFD2

UBE4B is implicated in K48-linked as well as K63-linked multiubiquitination. This protein is an E3 ligase with E4 activity and is best studied for its role in p53 polyubiquitination and degradation. Knockout of this gene results in embryonic lethality in mice and could be linked to stabilized p53. Ube4b^-/-^ mouse embryonic fibroblasts (MEFs) exhibit elevated levels of p53 and decreased p53 polyubiquitination [130, 133]. UBE4A, the lesser studied paralog of UBE4B shows E4 activity in DNA damage pathway [134]. In colon cancer, UBE4B levels are elevated even in precancerous lesions with highest expression levels in high grade dysplasia. As a negative regulator of p53 UBE4B may prove an important factor to study in carcinogenesis. PTBP3 modulated p53 levels by stabilizing UBE4A mRNA and enhancing cell proliferation in colorectal cancers, suggesting UBE4A and B have similar functions [135, 136]. A UBE4B homolog UFD2a is known to express two isoforms UFD2a7 and 7/7a and they are tissue specific. These isoforms are splice variants that differ in exon 7, the longer isoform has an additional exon (exon 7a). Further research is required to understand if these isoforms are expressed ubiquitously and if they have any role in carcinogenesis [137].

### Deubiquitinating Enzymes (DUBs)

Deubiquitinating enzymes (DUBs) are essential to replenish the free ubiquitin pool in cells, proofreading ubiquitin-protein conjugates, processing ubiquitin precursors and preventing the inhibition of the 26s proteasome by ubiquitin chains [138]. DUBs are part of the protease superfamily and are classified as either cysteine or zinc metalloproteases. Human DUBs are further classified into seven categories namely, Otubain proteases (OTUB), Ubiquitin specific proteases (USP), Ubiquitin C-terminal hydrolases (UCH), Machado-Joseph domain proteases (MJD), Jab1/Mov34/Mpr1/Pad1/MPN N-terminal (JAMM) domain metalloproteases, monocyte chemotactic protein-induced protease family (MINDYs), and Zn finger and UFSP domain proteins (ZUFSPs) [139].

#### OTUB family

This is an example with two isoforms (OTUB1 and 2) that differentially regulate substrate deubiquitination. Both these isoforms are overexpressed in gastric cancers when compared to normal tissues and OTUB1 levels are significantly higher than OTUB2 [140]. When 156 gastric cancer samples were studied to analyze the OTUB1 isoform 2 mRNA levels, it was found that OTUB1 isoform 2 is localized predominantly in the nucleus and showed significantly higher expression levels in gastric cancer samples. OTUB1 isoform 2 expression was associated with poorer survival, tumor growth and metastasis of gastric cancer in tumor xenograft models [141]. OTUB1 regulates RNF128/GRAIL, and thus is critical in T cell anergy. OTUB1 isoform 1 expressing bone marrow T cells from transgenic mice showed low levels of GRAIL, were proliferative and produced IL2, while cells expressing OTUB1 isoform 2 (splice variant) had high levels of endogenous GRAIL and were functionally anergic when stimulated with radiation [142]. Studying these ubiquitin specific proteases is of special interest to us as GRAIL contributes to mutant p53 stabilization in esophageal adenocarcinoma progression [14]. OTUB2 has a very similar function to OTUB1 [143]. In GI cancer samples (Fig. 1), we noted four OTUB1 and two OTUB2 isoforms, with moderate minor isoform frequency variation for OTUB1 and weak variation for OTUB2 and no pattern for either across the different cancer types.

#### USP Family

The USP9 subfamily consists of the two isoforms USP9X and USP9Y. Sixty-eight cases of patients with gastric cancer were analyzed for USP9X expression revealing that this protein is significantly overexpressed and linked with poor survival. On the other hand, USP9Y levels are high in normal tissues and low in tumor tissue suggesting that USP9X could be a potential oncogene [140, 144]. In triple negative breast cancer, inhibition of USP9X mediated NOTCH signaling using a small molecule resulted in decreased tumor growth without associated toxicity in a murine TNBC model [145]. USP9X deubiquitinates Histone lysine demethylase 4 C, overexpression of which is associated with poor prognosis and radioresistance in lung cancer and is associated with poor survival in non-small cell lung cancer, further supporting the possibility that USP9X is an oncogene [146, 147].

As paired, highly homologous pseudo-autosomal genes, with gender-specific expression and multiple isoforms, USP9X and USP9Y variant analysis would be complex and highly specialized. Cohort analysis would require substantial male and female presence, understanding of X-inactivation status of USP9X and strong mRNA sequencing data to continually identify specific isoforms. Thus USP9X & Y isoform analysis are beyond the scope of this review and as such were not included in Fig. 1.

#### MINDY Family

The family of Motif Interacting with Ubiquitin-containing Novel Deubiquitinating (MINDY) enzymes represent a newly discovered class of deubiquitinating enzymes that specifically target the removal of K48 linked ubiquitin. MINDY1, the first identified family member, is present in all eucaryotes [148] and has a strong bias for longer K48 linked Ub chains. A whole-genome, isoform-based RNAseq analysis of Barrett’s esophageal tissues (the precursor to EAC) demonstrated that *MINDY1* isoform variation was significantly associated with high grade dysplasia, but only when *TP53* mutation was present [149]. In this case, however the major isoform shifts from a protein producing one (ENST00000361936; MINDY1-203) to a non-coded isoform (ENST00000470877; MINDY1-204), potentially resulting in a reduction in MINDY1 protein levels. GTEx portal data indicate MINDY1-203 as one of three dominant isoforms seen across normal human tissues while the 5’ truncated MINDY1-204 is a minor isoform in studied normal tissues, except for the highly proliferative EBV transformed lymphocytes, which show stable MINDY-203, but a much-reduced level of protein forming MINDY1-204, suggesting that loss of the protein-coding MINDY1 might be associated with increased proliferation. Interestingly, these data contrast dramatically with findings in bladder and breast cancer, where MINDY1 was shown to be mitogenic via DUB activity on YAP and estrogen receptor alpha (ESR1), respectively [150, 151]. In TCGA tumor data from GI cancers, we noted that minor isoform frequencies for MINDY1 showed a similar tissue specific profile to those of MDM2/WWP2, where cancer types towards the upper GI (ESCC, EAC and stomach cancer types) showed minimal isoform variation, while the lower GI forms showed higher levels of variation (Fig. 1).

To better understand isoform-specific details of the above referenced UBS enzymes, we have added Supplemental Data, including isoform information along with attached links to UCSC browser (http://genome.cshlp.org/content/12/6/996.abstract). Utilization of these built-in UCSC Browser features will allow readers to analyze any gene-of-interest.

## Conclusion

In conclusion, we found minimal specific information showing isoform differences among UPS family members in GI cancers. However, there are examples (UBA1, UBCH5, RNF128/GRAIL, MDM2, BRCA etc.) and our analysis of isoform expression from the TCGA dataset suggests the importance of UPS related phenocopy changes in GI cancer progression and supports a need for further close analysis. To explore isoforms in other systems, users can consider:

1. I*soform screening systems*, focused on either systematic analysis of raw RNAseq data or the transcript utilization data provided as part of most RNAseq workflows (e.g., nf-core rnaseq pipeline: [152]). From RNAseq transcriptome data, and from some chip-based expression arrays (e,g., Affymetrix STGENE array) one specific set of analyses would be searching for relative changes in prominent isoform usage (isoform switching) in terms of a ratio ([153]; switchR [154], IsoformSwitchAnalyseR [155]). These software and approaches that look specifically at a switch in the prominent isoform utilization come with the advantages of: (a) providing one measure per multi-isoform gene expression and therefore don’t add to the type1 error problem seen in more gene-based differential analysis; (b) the potential for specific differential biology investigation, for example loss of a transmembrane domain or binding partner kinetics; and (c) the potential to extend to specific phenocopy genes, such as additional gene family members. As validation of these isoform changes will be essential for acceptance, protein-based mechanistic studies would not only improve acceptance but provide for future translational potential of such findings. This approach led to our understanding of RNF128 isoform differences in BE progression [14] and our subsequent ideas for mutant p53 targeting [15, 156].
2. *Biology-based isoform investigation*, as shown by our analysis of UBCH5A vs C in BE/EAC [15], following identification of E3 (RNF128) variants, we reasoned that characterization of the E2s involved in the cell-type specific interaction between RNF128 and mutant p53 (substrate) and led us to the UBCH5A vs UBCH5C paradigm, which turned out to be meaningful. As an example of this idea, following discovery of differential gene biology, a literature search for biologically pertinent isoform variations for the target gene and surrounding biology is a relevant approach. Tools that can be helpful for this include the TSVdb [11] online database which provides by-gene isoform utilization views for the TCGA cancer cohorts. Key features of this web-based application are the inclusion of normal samples, where possible, and the ability to do initial investigations into stage or survival-based data, although further TCGA independent validation of these details would be essential. Another source of genome-wide isoform utilization data is provided by the GTEX analysis across all available normal human tissues [4]. Differential tissue utilization, particularly involving a specific tissue or organ of interest, might provide a clue to interrogate certain gene expression, or protein variations in cancer cohort data.

### Supplementary information

Supplemental Figures

Supplemental Methods
