# Supplementary material for: Isoform alterations in the ubiquitination machinery impacting gastrointestinal malignancies
Source: Cell Death Dis. 2024 Mar 8;15(3):194. doi: 10.1038/s41419-024-06575-z (PMC10920915; doi:10.1038/s41419-024-06575-z)
Supplement: Supplementary file 1 — Supplemental Figures [file 41419_2024_6575_MOESM1_ESM.pdf]

GENCODE V44 hg38 Display Conventions and Configuration

Track displays the basic GENCODE set, splice variants, and non-coding genes.

Gene symbols, GENCODE Transcript ID (ENST00000561183.5) and UCSC Known Isoform ID (uc001yve.4).

The exons for coding open reading frames are thicker.

Coloring for the gene annotations is based on the annotation type:

- **coding**: protein coding transcripts, including polymorphic pseudogenes
- **non-coding**: non-protein coding transcripts
- **pseudogene**: pseudogene transcript annotations
- **problem**: problem transcripts (Biotypes of retained_intron, TEC, or disrupted_domain)

UniProt SwissProt/TrEMBL Protein Annotations Display Conventions:

Genomic locations of UniProt/SwissProt annotations are labeled with a short name for the type of annotation (e.g. "glyco", "disulf bond", "Signal peptide" etc.).

TrEMBL annotations are always shown in **light blue**, except in the Signal Peptides, Extracellular Domains, Transmembrane Domains, and Cytoplamsic domains subtracks.

The subtracks for domains related to subcellular location are sorted from outside to inside of the cell: **Signal peptide**, **extracellular**, **transmembrane**, and **cytoplasmic**.

In the "UniProt Modifications" track, lipoification sites are highlighted in **dark blue**, glycosylation sites in **dark green**, and phosphorylation in **light green**.

TCGA Cancer types:

- Colon adenocarcinoma
- Liver hepatocellular carcinoma
- Pancreatic adenocarcinoma
- Rectum adenocarcinoma
- Stomach adenocarcinoma
- Cholangiocarcinoma
- Esophageal carcinoma

TCGA Cancer types:

- Colon-Sigmoid
- Colon-Transverse
- Esophagus-Gastroesophageal Junction
- Esophagus-Mucosa
- Esophagus-Muscularis
- Liver
- Pancreas
- Small Intestine-Terminal Ileum
- Stomach

UBA1

multi-region chrX:47,193,829-47,215,128 4,783 bp.

UCSC Browser

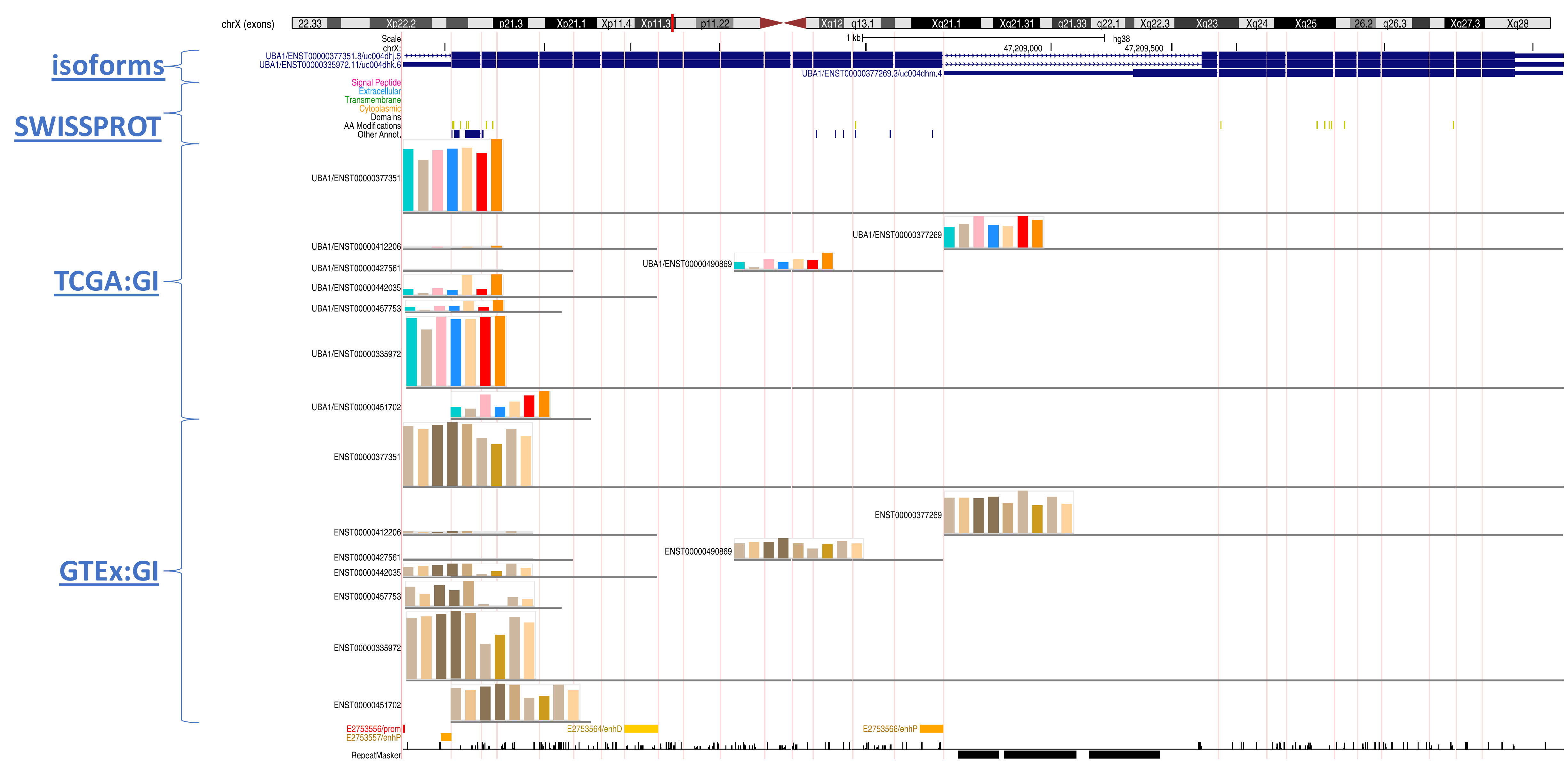

NOTE: This a manually downloaded and constructed PDF version – click the "UCSC Browser" button to see live track view

UBA6

multi-region chr4:67,612,652-67,701,155 10,258 bp.

UCSC Browser

isoforms

SWISSPROT

TCGA:GI

GTEx:GI

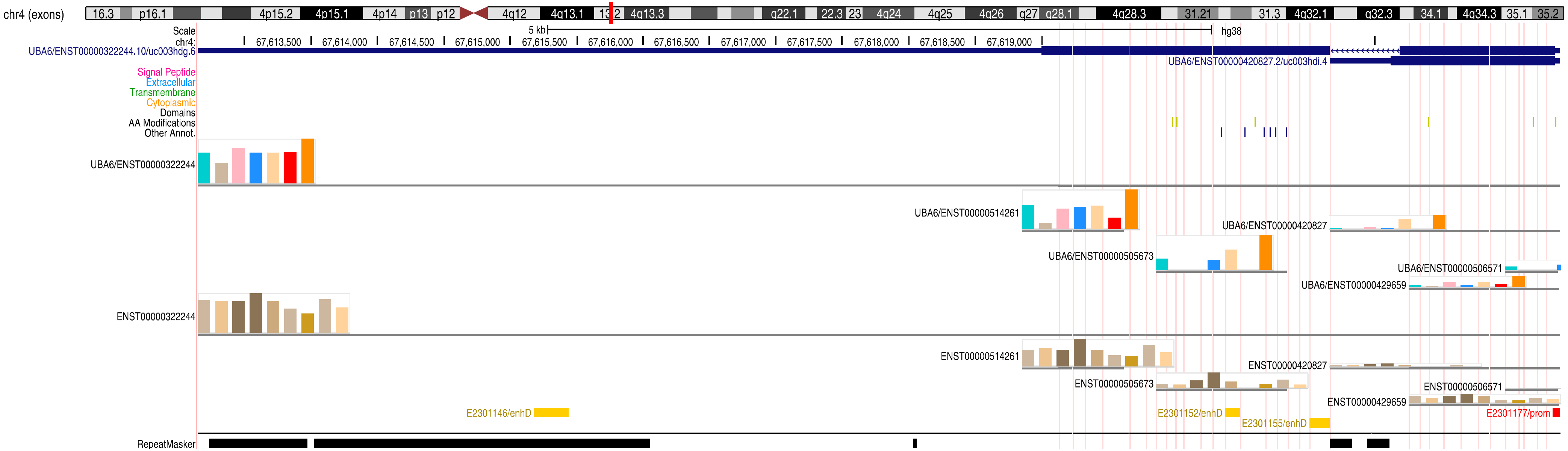

NOTE: This a manually downloaded and constructed PDF version – click the "UCSC Browser" button to see live track view

UBE2A1

multi-region chrX:119,574,514-119,583,280 851 bp.

UCSC Browser

isoforms

SWISSPROT

TCGA:GI

GTEx:GI

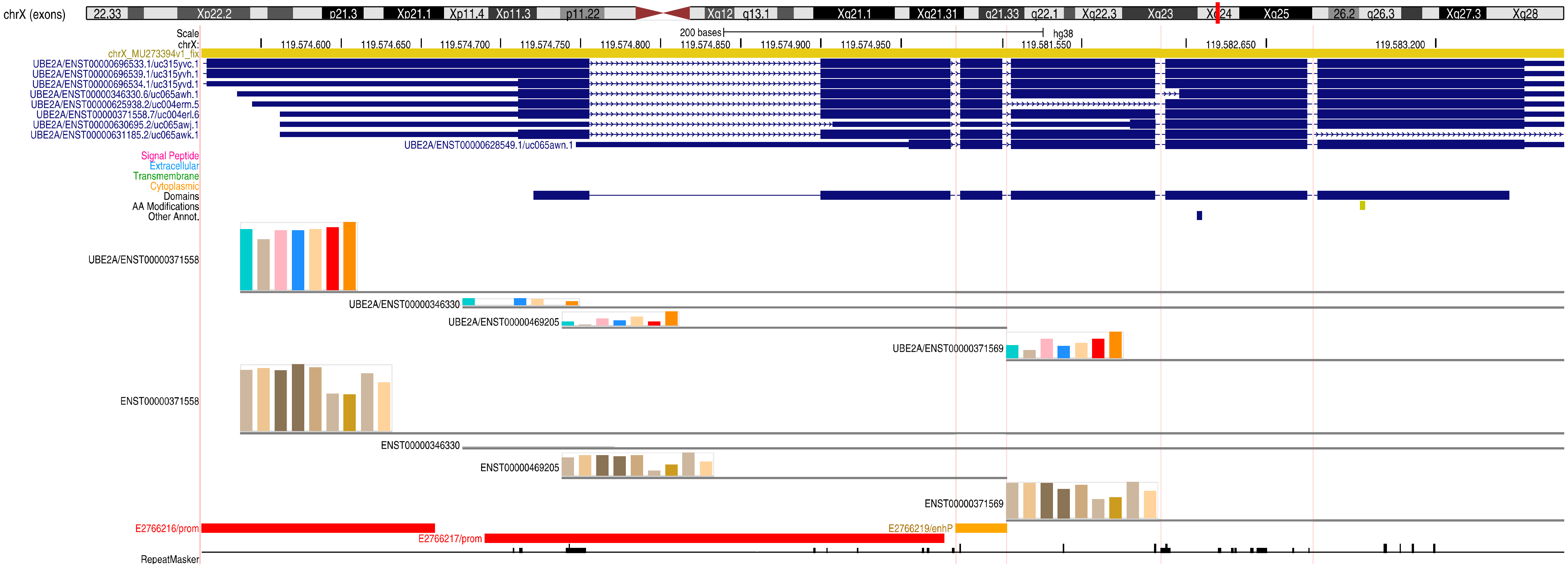

NOTE: This a manually downloaded and constructed PDF version – click the "UCSC Browser" button to see live track view

RNF128

multi-region | chrX:119,574,514-119,583,280 | 851 bp.

UCSC Browser

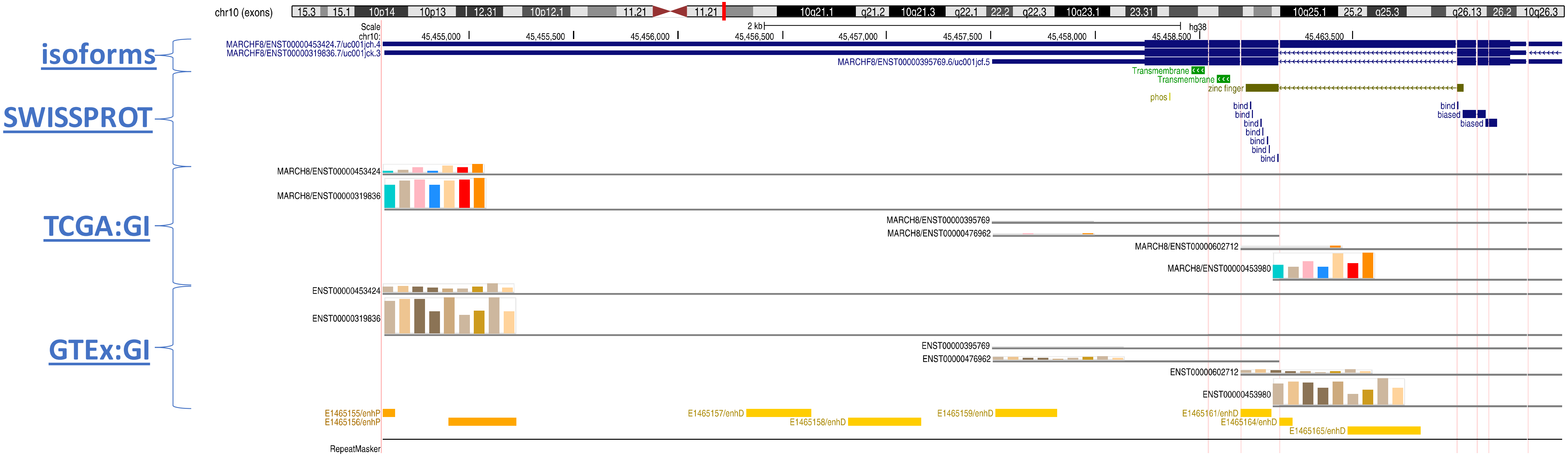

NOTE: This a manually downloaded and constructed PDF version – click the "UCSC Browser" button to see live track view

MDM2

multi-region chr12:68,808,370-68,839,918 1,953 bp.

UCSC Browser

isoforms

SWISSPROT

TCGA:GI

GTEx:GI

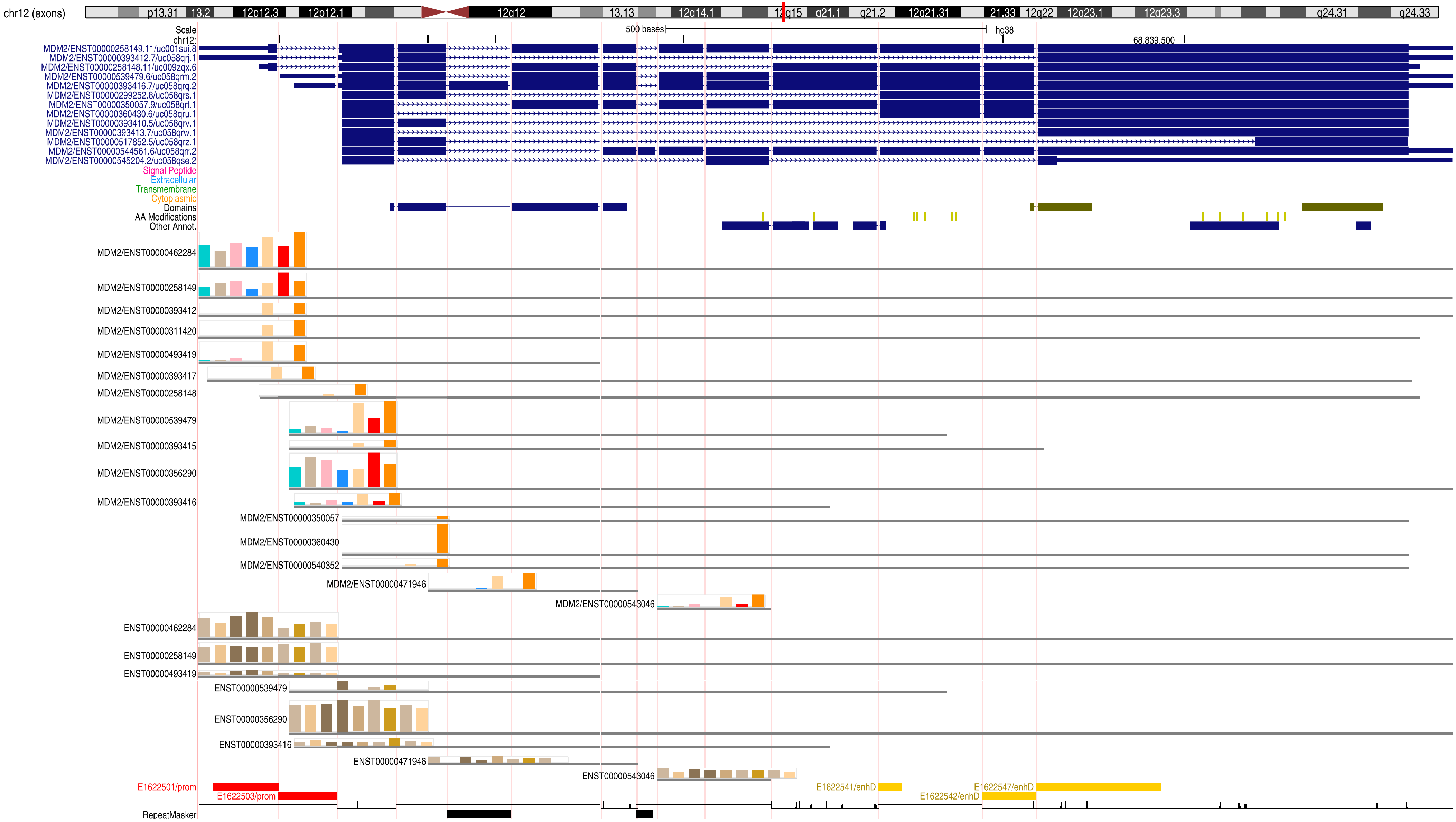

MDM4

multi-region chr1:204,516,509-204,549,752 1,826 bp.

UCSC Browser

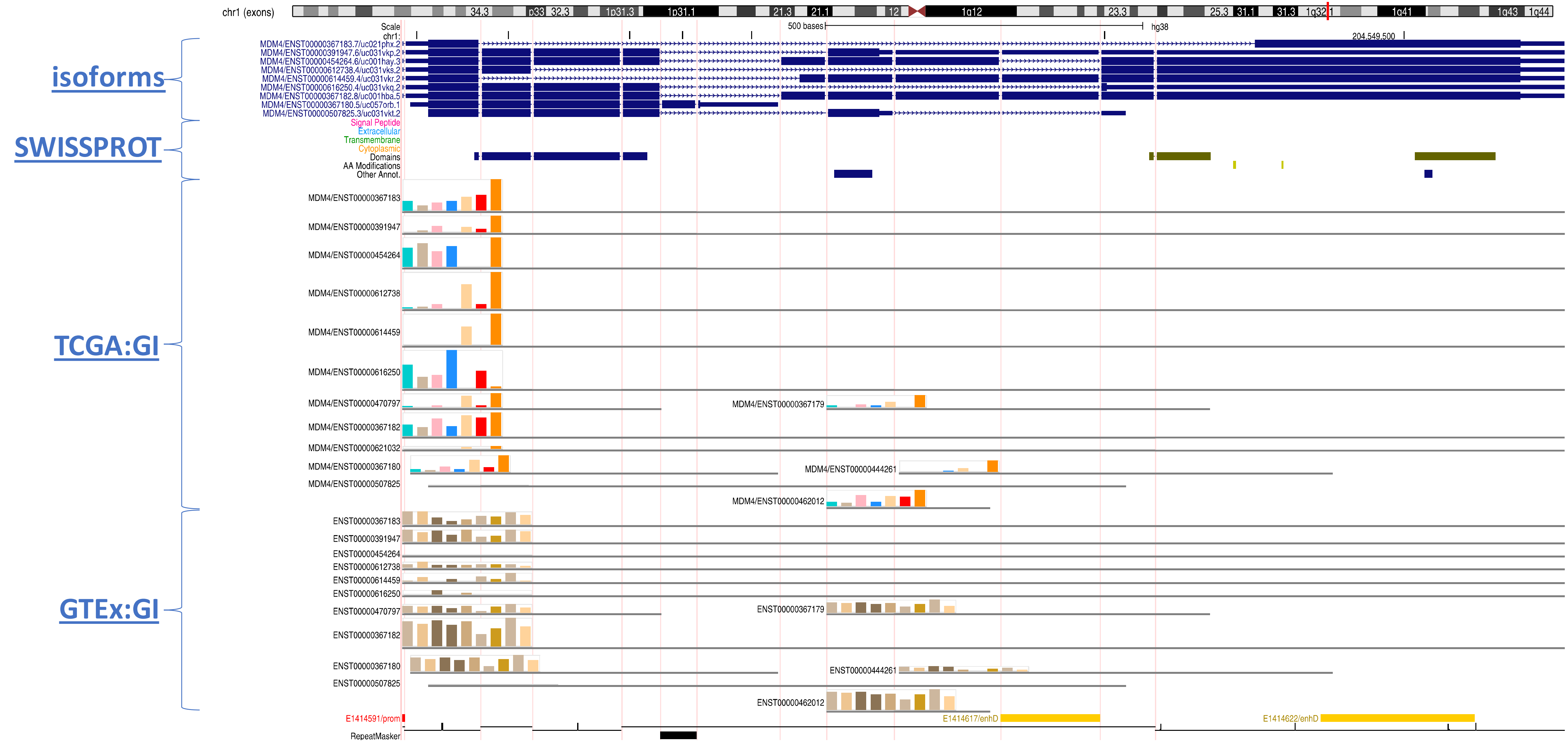

NOTE: This a manually downloaded and constructed PDF version – click the "UCSC Browser" button to see live track view

FBWX7

multi-region chr4:152,322,763-152,412,528 3,285 bp.

UCSC Browser

isoforms

SWISSPROT

TCGA:GI

GTEx:GI

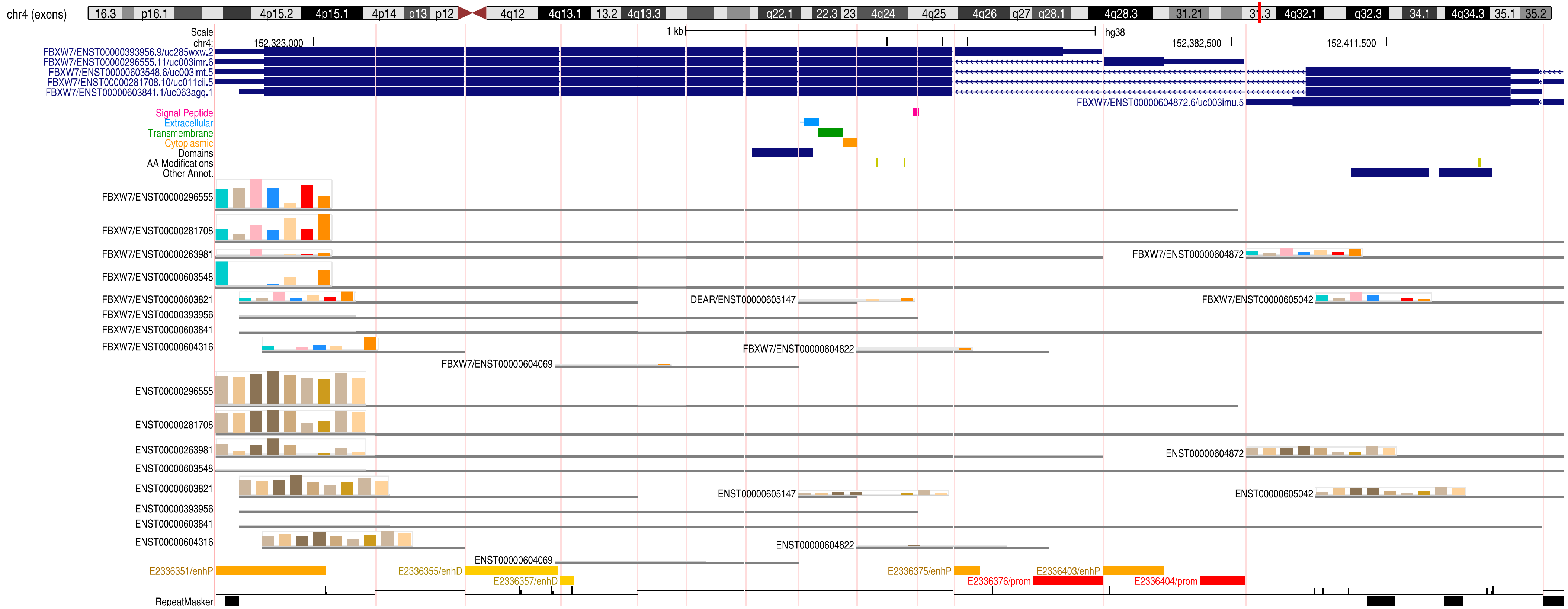

NOTE: This a manually downloaded and constructed PDF version – click the “UCSC Browser” button to see live track view

MARCHF8

multi-region | chr10:45,454,585-45,535,371 | 5,657 bp.

UCSC Browser

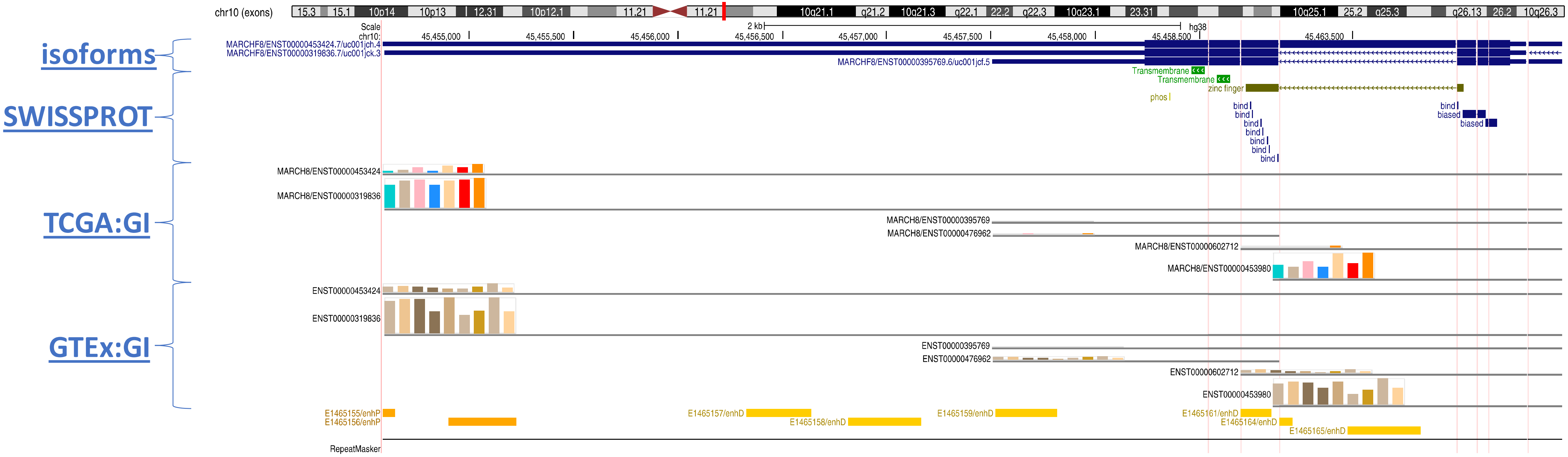

NOTE: This a manually downloaded and constructed PDF version – click the “UCSC Browser” button to see live track view

SIAH1

multi-region chr16:48,360,531-48,385,413 4,089 bp.

UCSC Browser

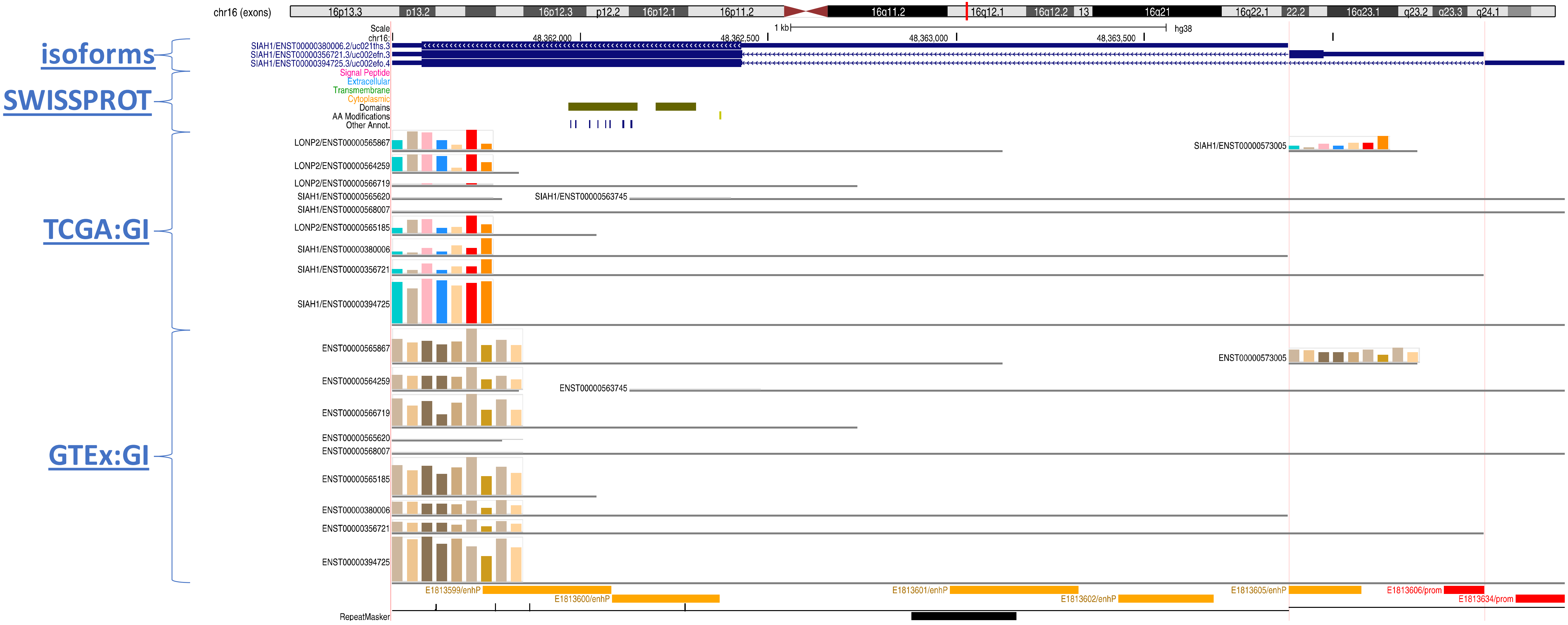

NOTE: This a manually downloaded and constructed PDF version – click the "UCSC Browser" button to see live track view

PELI3

multi-region chr11:66,466,905-66,476,300 2,435 bp.

UCSC Browser

isoforms

SWISSPROT

TCGA:GI

GTEx:GI

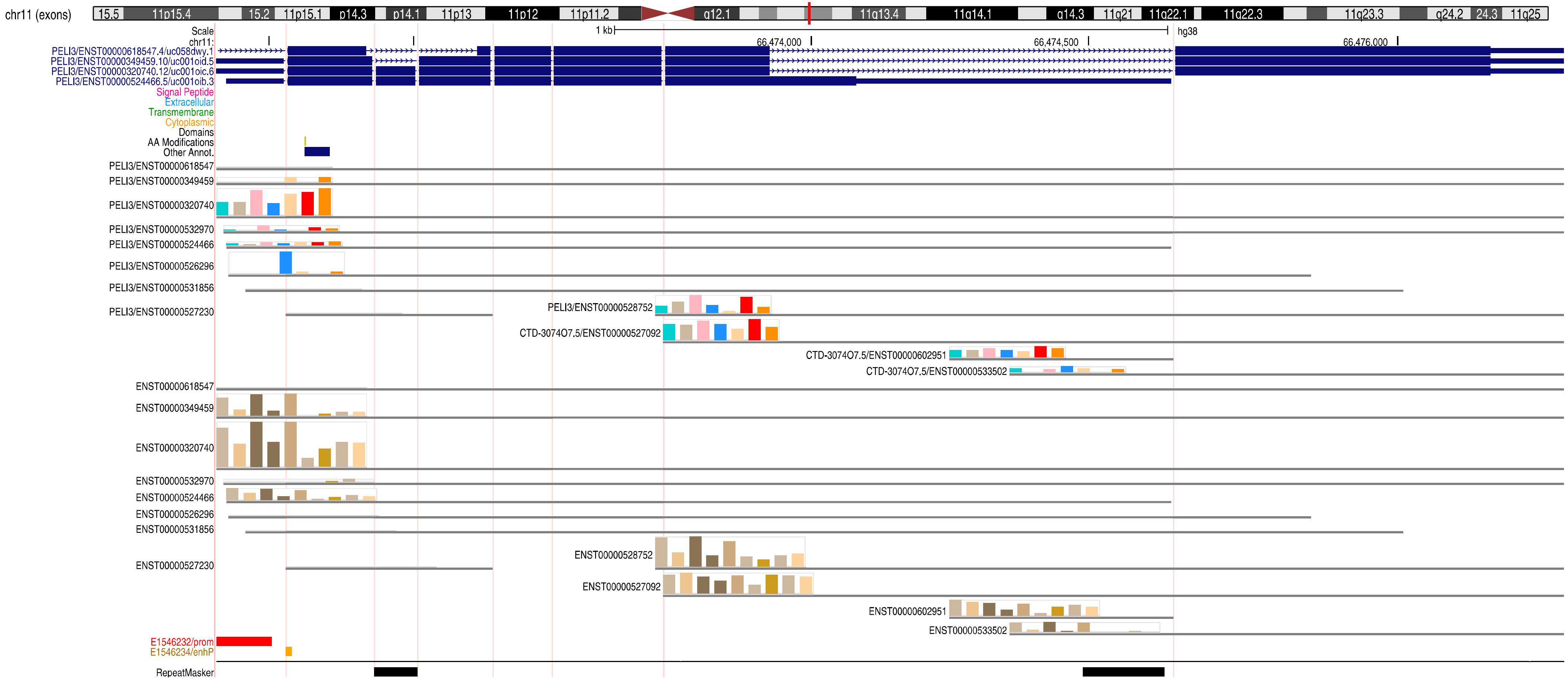

NOTE: This a manually downloaded and constructed PDF version – click the "UCSC Browser" button to see live track view

CBLB

multi-region chr3:105,658,600-105,869,012 4,312 bp.

UCSC Browser

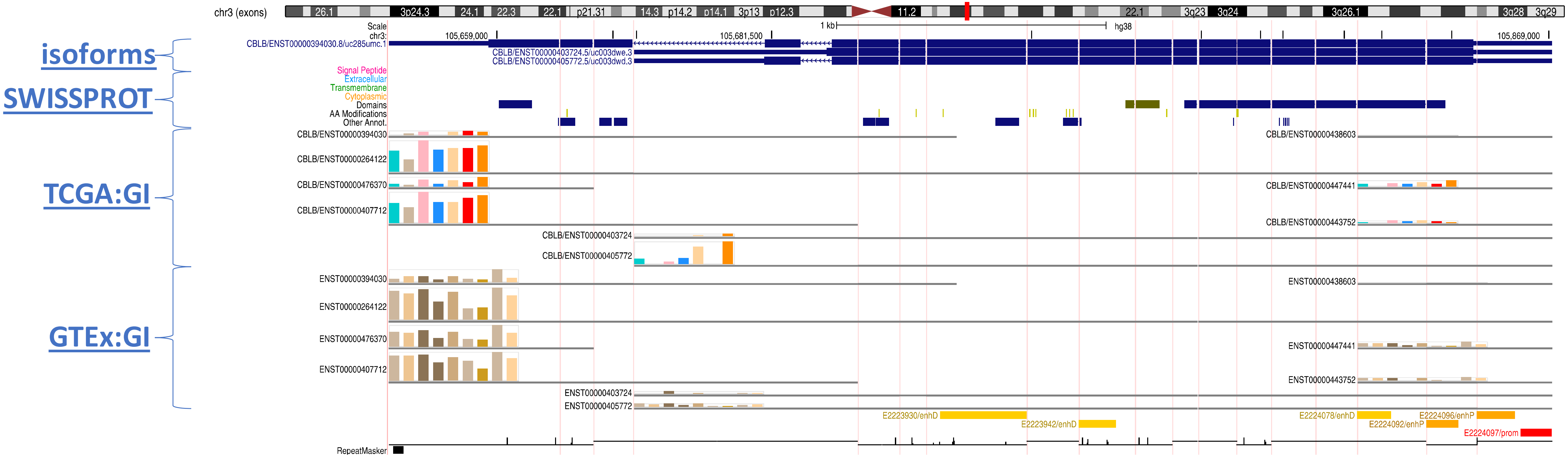

NOTE: This a manually downloaded and constructed PDF version – click the “UCSC Browser” button to see live track view

TRAF6

multi-region chr11:36,489,583-36,510,194 2,128 bp.

UCSC Browser

isoforms

SWISSPROT

TCGA:GI

GTEx:GI

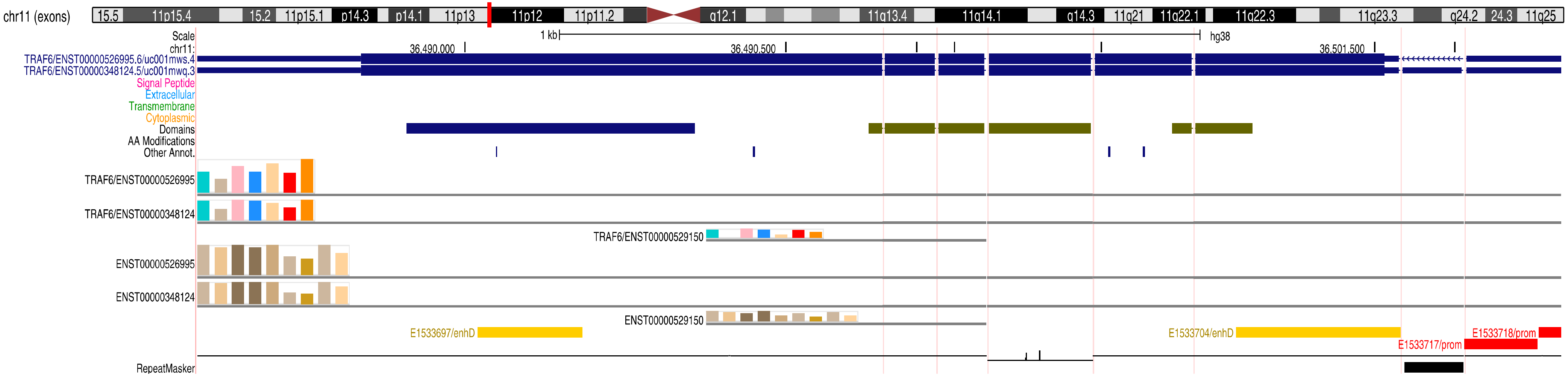

NOTE: This a manually downloaded and constructed PDF version – click the "UCSC Browser" button to see live track view

RNF43

multi-region chr17:58,353,676-58,417,534 6,197 bp.

UCSC Browser

isoforms

SWISSPROT

TCGA:GI

GTEx:GI

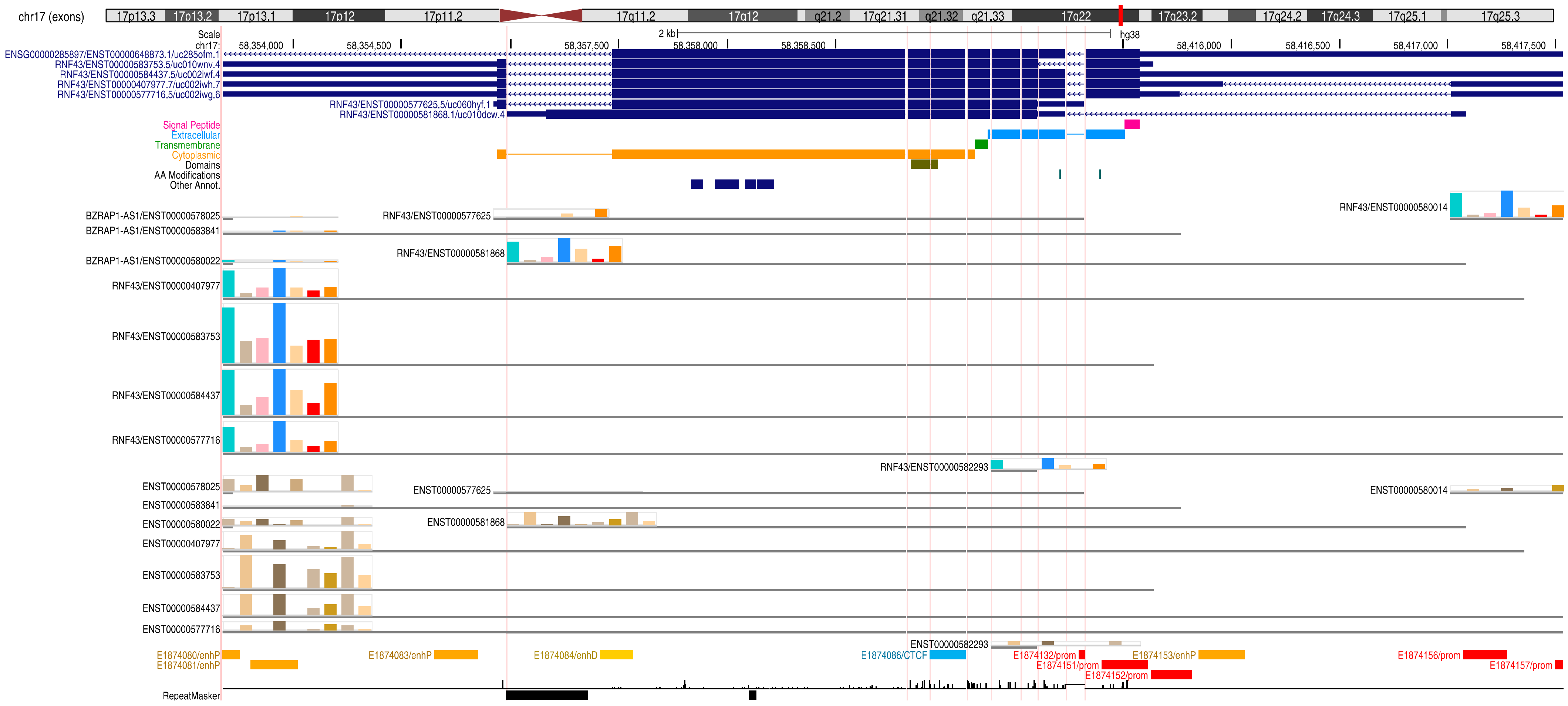

NOTE: This a manually downloaded and constructed PDF version – click the "UCSC Browser" button to see live track view

RBBP8

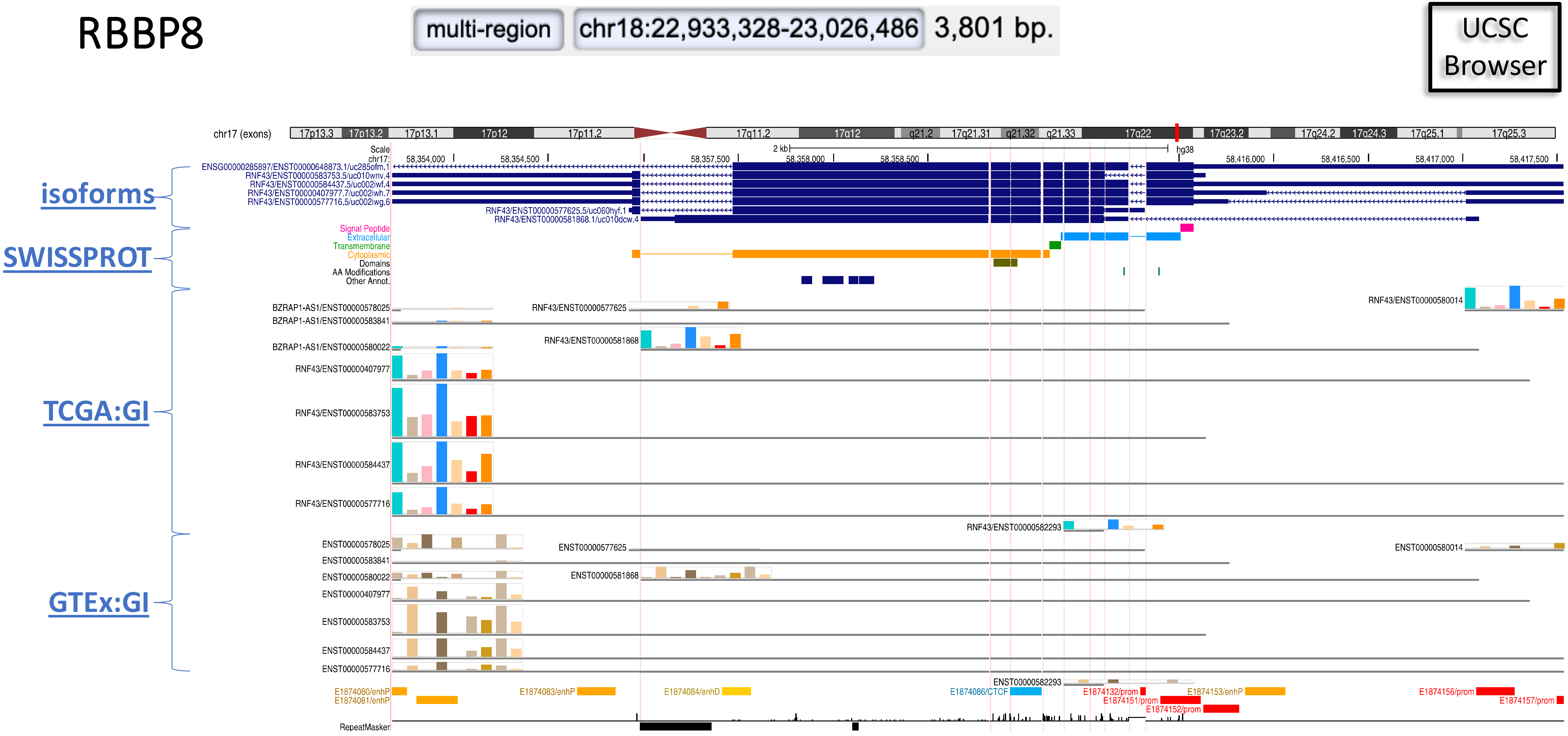

NOTE: This a manually downloaded and constructed PDF version – click the "UCSC Browser" button to see live track view

BRCA1

multi-region chr17:43,045,491-43,124,860 6,996 bp.

UCSC Browser

isoforms

SWISSPROT

TCGA:GI

GTEx:GI

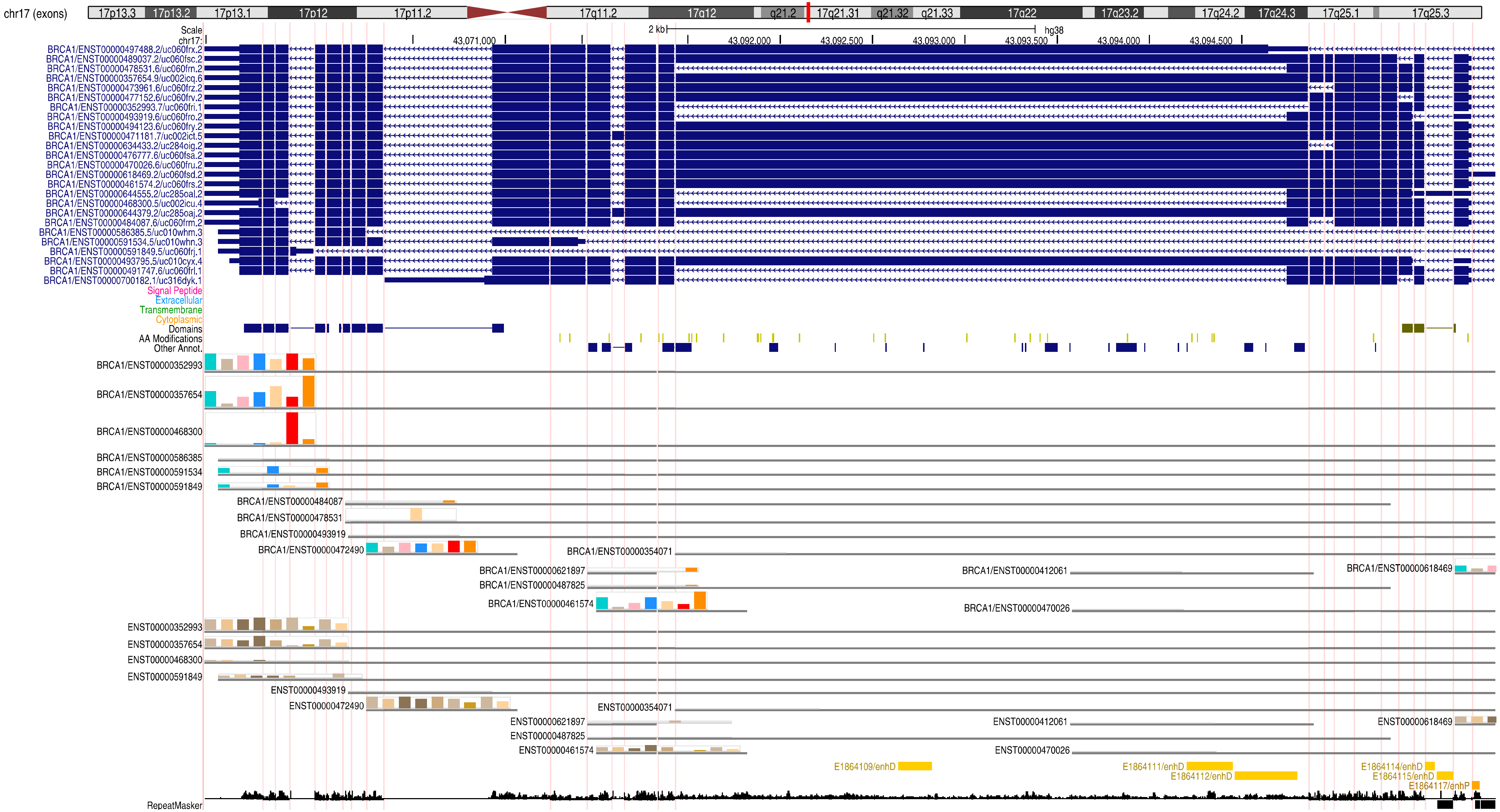

NOTE: This a manually downloaded and constructed PDF version – click the "UCSC Browser" button to see live track view

BARD1

multi-region chr2:214,728,541-214,809,634 2,594 bp.

UCSC Browser

isoforms

SWISSPROT

TCGA:GI

GTEx:GI

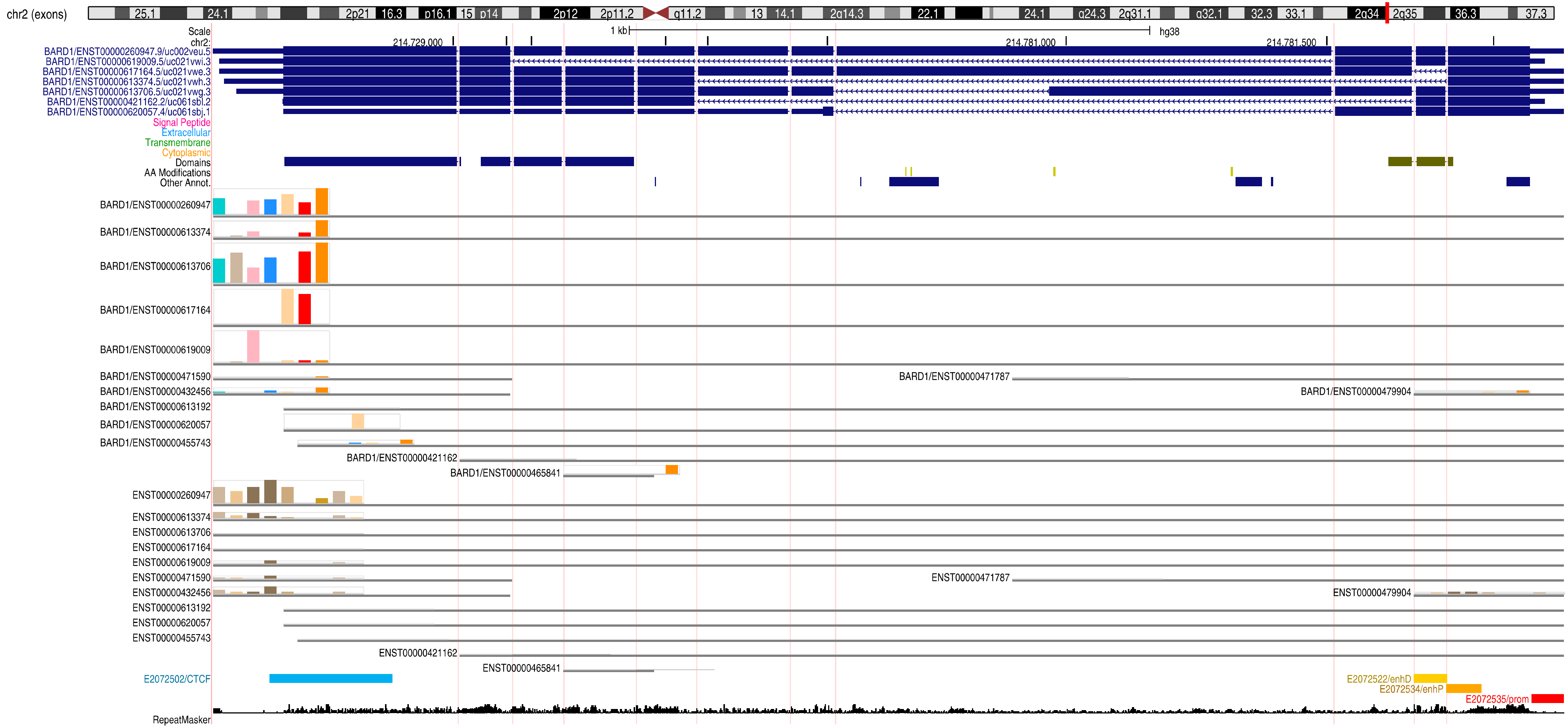

NOTE: This a manually downloaded and constructed PDF version – click the “UCSC Browser” button to see live track view

SMURF1

multi-region chr7:99,030,413-99,143,869 2,642 bp.

UCSC Browser

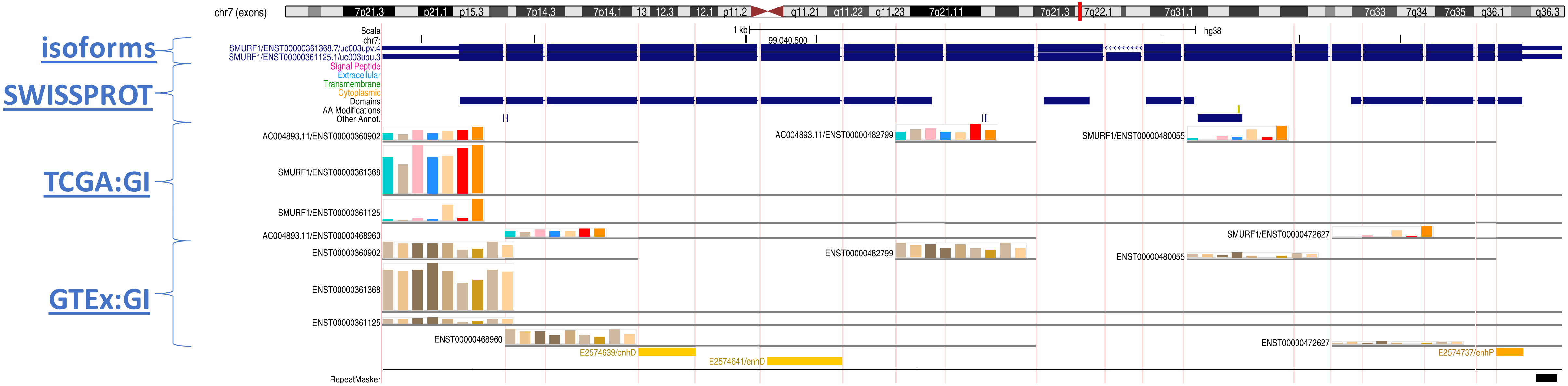

NOTE: This a manually downloaded and constructed PDF version – click the “UCSC Browser” button to see live track view

WWP2

multi-region | chr16:69,776,186-69,940,014 | 4,895 bp.

UCSC Browser

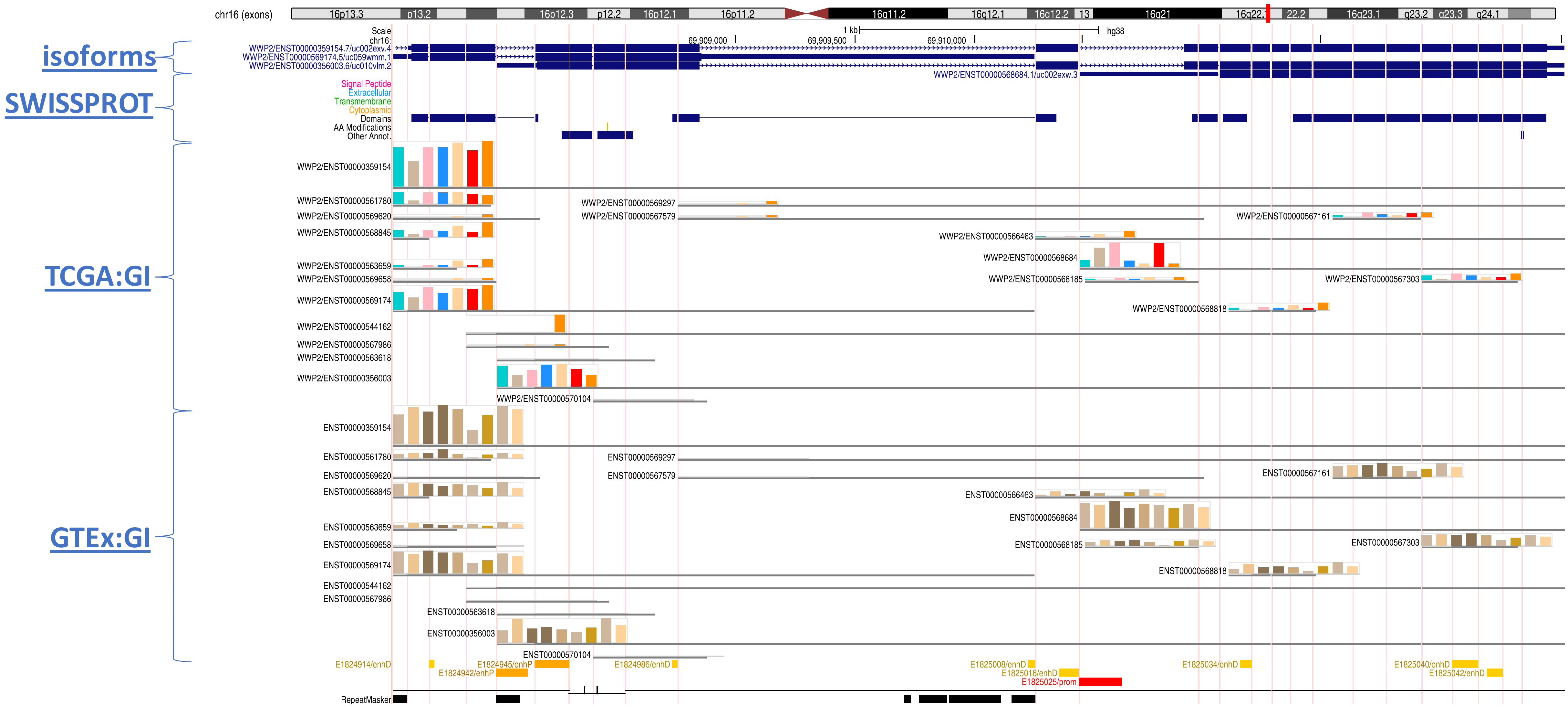

NOTE: This a manually downloaded and constructed PDF version – click the “UCSC Browser” button to see live track view

UBE4B

multi-region chr1:10,033,573-10,180,003 5,046 bp.

UCSC Browser

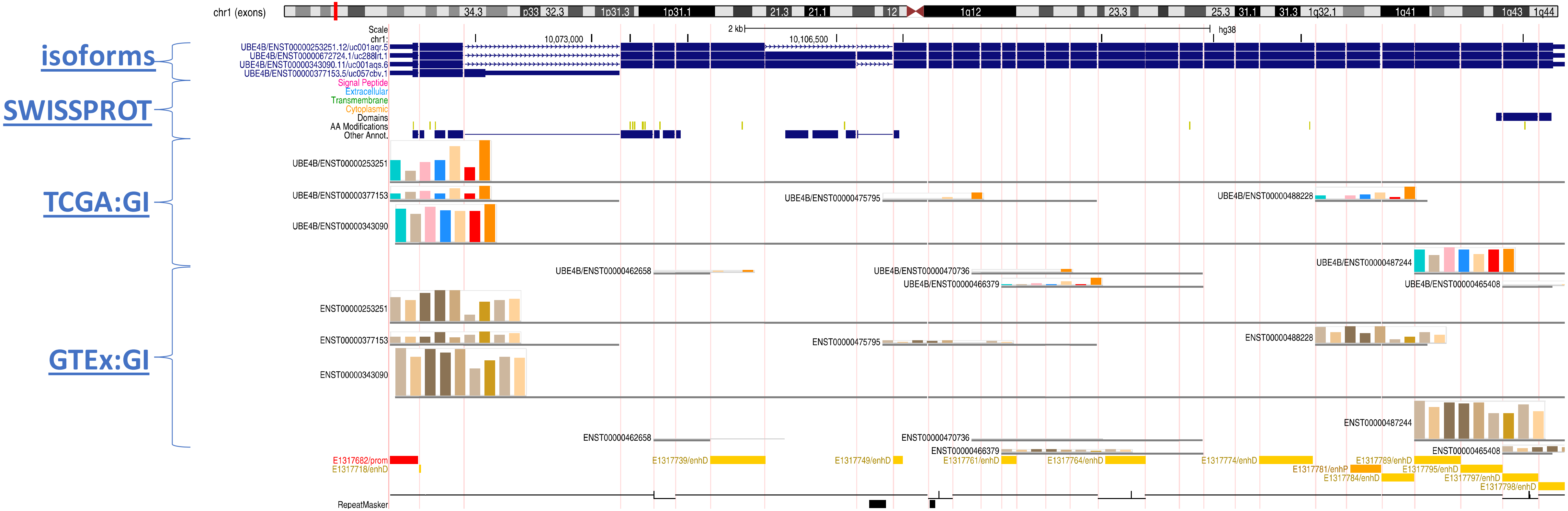

NOTE: This a manually downloaded and constructed PDF version – click the "UCSC Browser" button to see live track view

OTUB1

multi-region chr11:63,986,438-63,997,805 1,360 bp.

UCSC Browser

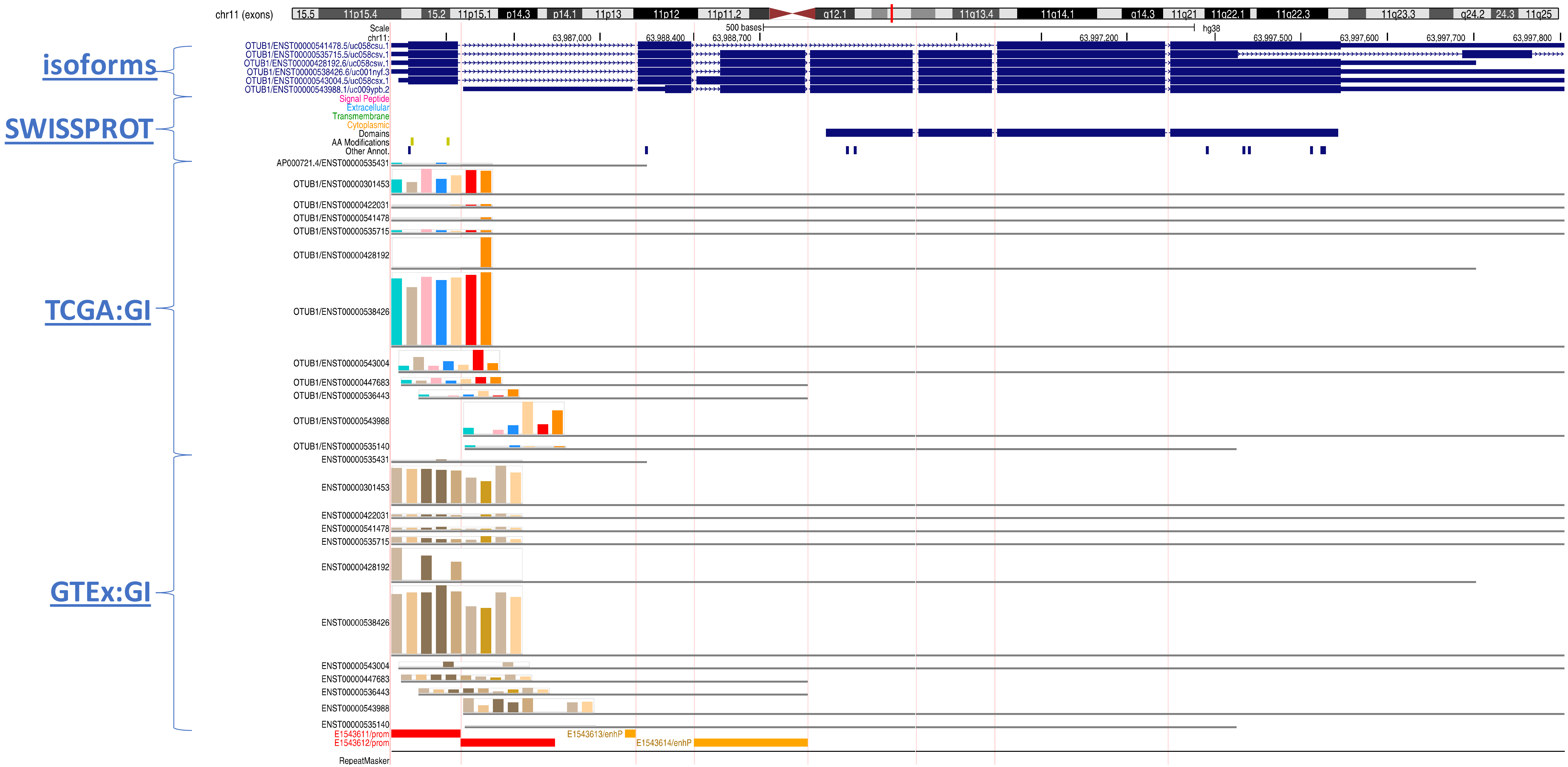

NOTE: This a manually downloaded and constructed PDF version – click the "UCSC Browser" button to see live track view

MINDY1

multi-region | chr1:150,997,242-151,002,722 | 1,608 bp.

UCSC Browser

isoforms

SWISSPROT

TCGA:GI

GTEx:GI

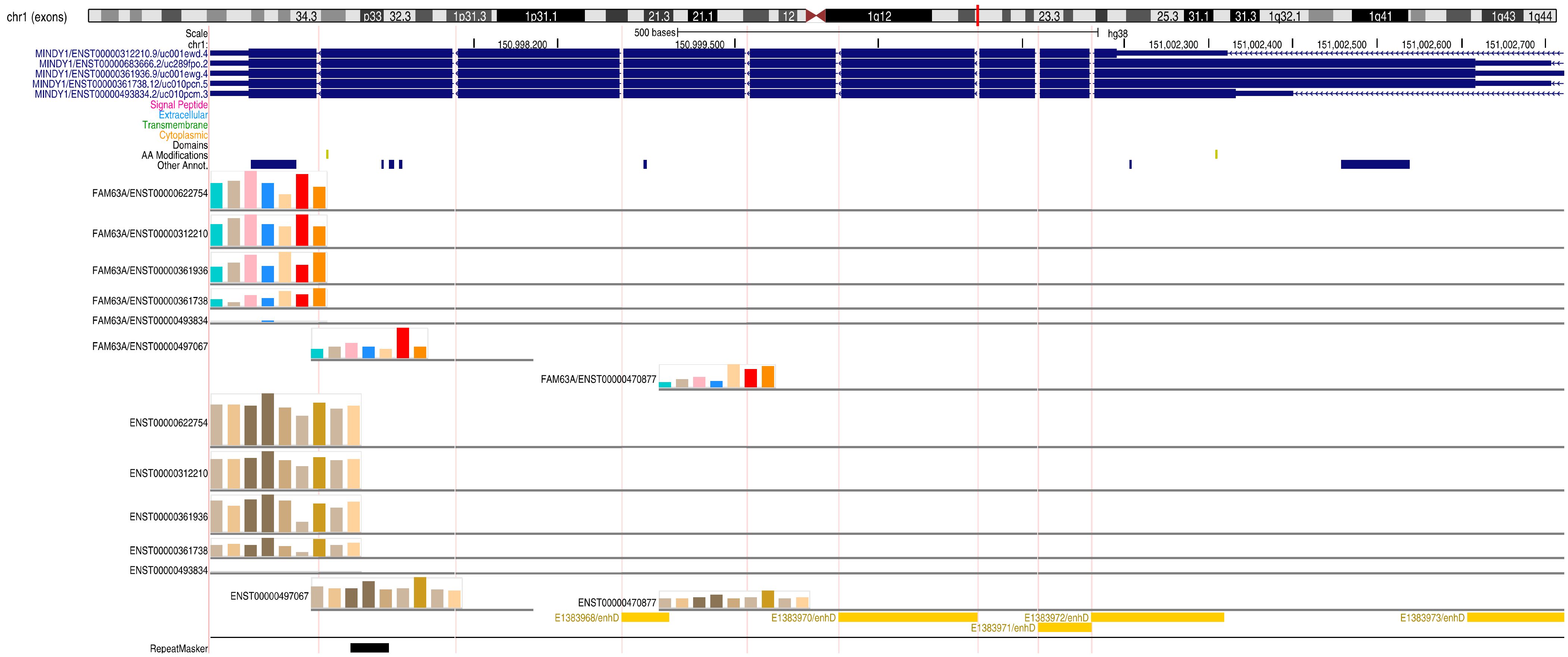

NOTE: This a manually downloaded and constructed PDF version – click the "UCSC Browser" button to see live track view
