# Supplementary material for: Isoform alterations in the ubiquitination machinery impacting gastrointestinal malignancies
Source: Cell Death Dis. 2024 Mar 8;15(3):194. doi: 10.1038/s41419-024-06575-z (PMC10920915; doi:10.1038/s41419-024-06575-z)
Supplement: Supplementary file 2 — Supplemental Methods [file 41419_2024_6575_MOESM2_ESM.pdf]

Supplemental Methods

For brevity and functional relevance, we restricted our isoform selection approach to those that resulted in alternate amino acid sequences. Isoforms result in other changes, such as promotor usage or untranslated region differences may also be important for cancer biology, but these are a) harder to identify and b) harder to study to catalogue cancer-related involvement.

1. **Pubmed GI literature search strategy**: We used the advanced PubMed search facility to craft a series of searches to identify GI cancer manuscripts that mentioned UBS-related genes by applying key words for cancer and UBS along with GI cancer-type specific key words as follows:

A) GI key word "OR" list : Anal, Bile duct, Colon, Esophageal, Oesophageal, Gallbladder, Liver, Pancreatic, Rectal, Intestinal, intestine, Stomach, Gastric, Gastrointestinal [resulted in 2,653,570 PubMed records] AND

B) Cancer key words: cancer, tumor, tumour [we tested each of these key words separately with sets A and set C, and the number of records (8736) was the same: so we used cancer [to 805,281 PubMed records] AND

C) Ubiquitin ("ubiq*"), star represents any remaining characters to the word beginning with 'ubiq' AND [to 266 PubMed records]

We found that a majority (8,263/8,736) of GI cancer papers included "protein", while a lower fraction include "gene", 3,861/8,768. In order to link with TCGA gene-based data, we need to link via gene symbol, so we did not include either of these terms and instead chose to search for mention of each identified UBS gene.

We then worked through the resulting 266 abstract records to confirm mention of one of the 427 UBS genes we identified. We sourced from several different locations including the human E3 Ubiquitin Ligases list (https://esbl.nhlbi.nih.gov/Databases/KSBP2/Targets/Lists/E3-ligases/) published in [DOI: 10.1152/physiolgenomics.00031.2016], lists from our previous UBS review and published reviews on E1 and E2 genes. When conducting gene searches (based on gene symbol), we noted that:

a. Some gene families required individual gene symbol searches to restrict false positives: examples include MID1&2, PELI1-3 and PLAG1.
b. Some genes which had undergone symbol changes required searches with both symbols, for example MARCH family members now share the prefix MARCHF.
c. In some cases, not all members of gene families are considered part of the UBS and thus known UBS members needed to be search for individually, include members of the BIRC, SMURF, HERC, UBR, PCGF, MKRN and TRIM gene families.
d. Some genes, such as ITCH and UNK gave a significant number of false positives.

With these characteristics in mind, a majority of genes were searched by the shared portion of the gene family, followed by star, e.g., FBWX*.

We filtered and ranked papers GI cancer-associated, UBS-related papers and screened abstract records for those that appeared to mention one of the 427 UBS-related genes. We highlighted the abstract or manuscript (if needed) section to see whether it mentioned isoforms for the identified UBS gene(s), and whether the tone of the manuscript indicated whether isoform variation was associated with clinical characteristics. This resulted in a total of 35 papers, that were graded from clear, moderately clear, or suggestive, depending on the context of UBS gene isoform mentions. These papers were then screened by us for relevance, which resulted in the identification of 25 papers.

2. **Protein-base literature search**: A protein biochemist screened GI-cancer papers via key words to those that mentioned UBS genes or proteins and investigated the context, looking for evidence that protein isoforms were involved in the reported GI-cancer outcomes.

3. **Expert UBS gene additions**: Our team has >20 years of experience understanding the role of post-translational protein modification in cancer cell biology, added a core set of genes (WWP1, WWP2, SMURF1, SMURF2 etc.) each with a history of cancer involvement, but not identified by the above screens.

4. **Combine list search**: Publication lists, removed duplicates, then screened and recorded text that indicated isoforms were associated with disease progression, initiation, or patient outcomes. Through this three-pronged screening system we hoped to identify a core set of UBS protein for GI cancer consideration.

5. **Extract GI clinical data**: GI organ specific clinical data was extracted for TCGA cohorts using the cBioportal interface and imported into EXCEL. Key fields were aligned between cancer cohorts and samples were ordered into subgroups based on "Cancer, Detailed" subtype entry. Cancer subtypes with more than 10 samples were included in Tables I & II. In addition, a series of non-cancer (normal) tissue samples from cancer patients were isolated as a reference group (n=160). This reference/non-cancer subset was comprised a varying number from each organ-based cohort (ESCA (11), STAD (35), LIHC (50), PAAD (4),CHOL (9) & COADREAD (51).

6. **Extract GI gene/isoform data**: Genes identified with GI cancer specific evidence from step 5, had isoform data and gene-structure pattern data manually extracted from the TVSdb interface and organized, per-gene into a single spreadsheet with a sample order matching clinical subgroups in step 6.

7. **Isoform frequency and diversity calculations:** Sample data for isoforms in each TCGA GI cohort were tallied to determine the number of isoforms observed, per gene, in each pathology subtype (frequency < 0.01), and overall, across the combined GI cancer cohort. This was contrasted against normal, organ-wide protein atlas data.
   Using the combined non-cancer sample group as a reference, three distinct diversity measures were derived for each histological subtype, for each gene: Shannon Diversity Index, Shannon Equitability Index and minor allele frequency. Minor isoform frequency was described as the combined frequency of all but the most common isoform observed in the combined GI cancer cohort.
   Using correlations across pathology subtypes, we found (sub table X and Y) that minor isoform (the simplest to calculate and describe - most direct) where highly correlated with both diversity measures and hence chose this as our reporting variable used in Tables 1 & 2.

8. **Identifying genes with diversity differences between cancer pathology groups and non-cancer pool**: By contrasting to the combined non-cancer sample subgroup, we identified genes were diversity in specific (or all) pathologic cancer subtypes deviated from the non-cancer control group. Thus, in Tables 1 and 2 we report on three measures for genes, the number of alleles seen, the minor allele frequency and indication of deviation from control minor allele frequency. Genes with a high minor isoform frequency could be seen as those where isoform variation had more opportunity to play a role in cancer processes. Due to the strong potential for complex relationships between isoforms and specific cancer outcomes, we specifically have not indicated -at risk isoforms in TCGA data. Depending on the data and gene circumstances, specific experiments would be required to solidify links between isoform expression patterns and cancer variables, which is beyond the scope of this review and authors. However, linked to gene names (click to open link) in Tables 1 and 2 and indicated in the text, we provide views from a custom UCSC Browser view which shows the per isoform exon representation, both NCBI and Ensembl isoform identities, and a link to any OMIM info on clinically relevant variants.
